# *CYP2C19* variability and clinical outcomes of clopidogrel, proton pump inhibitors, and voriconazole in Southeast Asia: a systematic review and meta-analysis

**DOI:** 10.3389/fphar.2025.1572886

**Published:** 2025-06-19

**Authors:** Harri Hardi, Agian Jeffilano Barinda, Liganda Endo Mahata, Zahra Fitrianti

**Affiliations:** ^1^ Clinical Pharmacology Specialist Study Program, Faculty of Medicine, Universitas Indonesia, Jakarta, Indonesia; ^2^ Department of Pharmacology and Therapeutics, Faculty of Medicine, Universitas Indonesia, Jakarta, Indonesia; ^3^ Metabolic, Cardiovascular, and Aging Cluster, Indonesia Medical Education and Research Institute (IMERI), Faculty of Medicine, Universitas Indonesia, Jakarta, Indonesia; ^4^ Department of Pharmacology and Therapy, Faculty of Medicine, Universitas Andalas, Padang, West Sumatra, Indonesia

**Keywords:** CYP2C19, Southeast Asia, clopidogrel, proton pump inhibitors, voriconazole

## Abstract

**Introduction:**

Polymorphism in *CYP2C19* is more prevalent in East Asia than in other regions of the world. However, no systematic review has analysed *CYP2C19* variation in the Southeast Asian population. The understanding of variation may serve as a foundation for pharmacogenetic research and testing in this domain. Therefore, this meta-analysis aims to clarify the variability of *CYP2C19* in Southeast Asia and its clinical implications for several medications, including clopidogrel, proton pump inhibitors (PPIs), and voriconazole.

**Methods:**

This systematic review employed the keywords “*CYP2C19*” and “Southeast Asia country,” obtained from PubMed, Scopus, Web of Science, and Google Scholar. Single proportion meta-analyses of allele distribution were performed utilizing inverse variance analysis. Meta-analyses of clinical outcomes were performed using the Mantel-Haenszel method.

**Results and discussion:**

Based on 72 found studies, this meta-analysis revealed that *CYP2C19* allele distribution in Southeast Asians is predominantly similar to that in East Asians, except for Indian Singaporeans and Malaysians, who match South and Middle Asians, and Papuans, who are similar to the Oceania population. *CYP2C19* variation in Southeast Asian populations correlates with different treatment responses to clopidogrel and PPIs. The incidence of major adverse cardiovascular events (MACE), hypoaggregation, and clopidogrel resistance increased among clopidogrel users with *CYP2C19* intermediate and poor metabolizer phenotypes. The effectiveness of PPIs treatment for Helicobacter pylori also tends to decrease in normal and intermediate metabolizers compared to poor metabolizers. Additional high-quality studies, including RCTs of pharmacogenetic testing, are essential to encourage *CYP2C19* testing in Southeast Asia.

**Systematic review registration:**

https://www.crd.york.ac.uk/prospero/display_record.php?RecordID=593116, CRD42024593116

## 1 Introduction

Southeast Asia consists of more than 25,000 islands, with populations migrating to Island Southeast Asia (ISEA) approximately 4,000 years ago. While the predominant theory posits that modern Southeast Asians trace their origins to Aboriginal Taiwanese, human genetic diversity in Southeast Asia is primarily divided by the Wallace Line, which demarcates mainland Southeast Asia and western Indonesia from eastern Indonesia (Hill et al., 2007). During the historical period, the genetic diversity of Southeast Asian populations was altered by migrations from China, India, and Middle Eastern countries (Karafet et al., 2010).

Genetic variation in drug-metabolizing enzymes appears to be closely associated with human populations and races (Nebert and Menon, 2001). This variance resulted in the development of the pharmacogenomics concept, which evaluates the role of genomics in drug response. Utilizing the proper medication and dosage according to genetic profiles would enhance therapeutic drug levels, improve drug effectiveness, and reduce adverse effects (Weinshilboum and Wang, 2017). Despite the involvement of numerous genes, the cytochrome P450 superfamily of enzymes, which includes *CYP2C19*, is responsible for about 75 percent of the pharmacological responses associated with routinely prescribed drugs (Chambliss and Chan, 2016).

A study involving 141,614 individuals globally revealed that *CYP2C19* poor metabolizers constituted 15% of the East Asian population, in contrast to merely 3% in Europe (Zhou and Lauschke, 2022). However, this study did not analyze the Southeast Asian population subgroup. In addition, most guidelines regarding the utilization of *CYP2C19* testing are primarily based on research conducted in developed countries. For instance, the two principal meta-analyses (Galli et al., 2021; Pereira et al., 2021) that supported the CPIC (Clinical Pharmacogenetics Implementation Consortium) guidelines regarding *CYP2C19* testing in clopidogrel (Lee et al., 2022) included no studies from Southeast Asia.

Clopidogrel is essential to antiplatelet therapy in cardiovascular disease, accounting for 37.9% of all-cause mortality in Southeast Asia (Khetan et al., 2024). Clopidogrel is also used for ischemic stroke, which constitutes 80% of all stroke types in Southeast Asia. The prevalence of stroke in Southeast Asia varied by country, with Singapore exhibiting the highest rate at 3.65% and Indonesia the lowest at 0.25% (Ng et al., 2019). Recently, the National Institute for Health and Care Excellence (NICE) released its first genotype testing recommendations regarding clopidogrel administration in ischemic stroke or transient ischemic attack (NICE, 2024), increasing the awareness of the utilization of *CYP2C19* genotype testing in other areas, including Southeast Asia.

This meta-analysis aims to demonstrate the demographic prevalence of *CYP2C19* polymorphism across countries and diverse ethnicities in Southeast Asia. This demographic prevalence aims to serve as a basis for subsequent pharmacogenetic research and testing in Southeast Asia. This study also aims to determine whether *CYP2C19* phenotype variation in the Southeast Asian population influences the effectiveness and safety of drugs metabolized by *CYP2C19*, thereby guiding clinical practices concerning the implementation of *CYP2C19* testing in the region.

## 2 Materials and methods

This systematic review was conducted in accordance with PRISMA guidelines (Page et al., 2021). The study protocol is available on The International Prospective Register of Systematic Reviews (PROSPERO) database under registration number CRD42024593116 (https://www.crd.york.ac.uk/prospero/display_record.php?RecordID=593116).

### 2.1 Study eligibility criteria

This systematic review included cross-sectional, case-control, cohort, and randomized controlled trials (RCTs). Studies were included if they analyzed *CYP2C19* variations in Southeast Asian populations. We excluded studies involving individuals of Southeast Asian descent residing outside Southeast Asia. The exposure and comparator of this systematic review are variations in *CYP2C19*. We predominantly acquired variants *2, *3, and *17. However, we also integrated additional variations that have been evaluated in populations. The outcome pertained to the efficacy and safety of drugs metabolized by *CYP2C19*. We also obtained the frequencies of each allele and the *CYP2C19* phenotype.

### 2.2 Search strategy and study selection

We conducted a systematic search across three databases: PubMed, Scopus, and Web of Science, from their inception until 4 October 2024, utilizing the keywords “*CYP2C19*” and “Southeast Asia country.” The countries of Southeast Asia are defined as those that are members of ASEAN (The Association of Southeast Asian Nations). We also conducted a search on Google Scholar using the same keywords, incorporating the first 50 studies from the search results of each country. No language restrictions were applied. Supplementary Table S1 presents a comprehensive utilization of keywords. Two independent authors (LEM and ZF) independently select suitable studies based on inclusion and exclusion criteria. Additional discrepancies were addressed with the third author (HH).

### 2.3 Data extraction and quality assessment

We obtained data regarding the study demographics (country and ethnicity), total subjects, and *CYP2C19* allele frequency and phenotype. We used classification derived from the Pharmacogenomics Knowledgebase (PharmGKB) *CYP2C19* Diplotype-Phenotype Table (https://www.pharmgkb.org/page/CYP2C19RefMaterials). This classification categorizes *CYP2C19* phenotypes into five primary groups: ultra-rapid metabolizer (UM), rapid metabolizer (RM), normal metabolizer (NM), intermediate metabolizer (IM), and poor metabolizer (PM). Studies utilizing outdated classifications, such as extensive metabolizer (EM), were categorized according to genotype in relation to phenotype classification.

We additionally extracted data on the analysis of *CYP2C19* phenotype variation in relation to drug effectiveness and safety. Table 1 includes the responses to clopidogrel, PPIs, and voriconazole associated with variations in the *CYP2C19* phenotype based on CPIC (Moriyama et al., 2017; Lima et al., 2021; Lee et al., 2022) and Dutch Pharmacogenetics Working Group (DPWG) guidelines. DPWG guidelines for each drug were extracted from PharmKGB clinical guideline annotations (https://www.pharmgkb.org/guidelineAnnotations). From all included studies, we extracted the drug dosage, study type, subject characteristics, and effectiveness or safety outcomes. Two independent authors (LEM and ZF) extracted the data, and discrepancies were deliberated with the third author (HH). Two independent authors (LEM and ZF) conducted a risk of bias assessment utilizing the Joanna Briggs Institute (JBI) critical appraisal tool. Additional discrepancies were addressed with the third author (HH).

**TABLE 1:** Clinical Pharmacogenetics Implementation Consortium (CPIC) and Dutch Pharmacogenetics Working Group (DPWG) guidelines for clopidogrel, proton pump inhibitors (PPIs), and voriconazole based on CYP2C19 phenotypes. The CPIC guidelines encompass PPIs such as omeprazole, lansoprazole, pantoprazole, and dexlansoprazole, while the DPWG guideline only addresses omeprazole, lansoprazole, and pantoprazole.

Drugs	Ultra-rapid metabolizer (UM)	Rapid metabolizer (RM)	Normal metabolizer (NM)	Intermediate metabolizer (IM)	Poor metabolizer (PM)
	*17/*17	*1/*17	*1/*1	*1/*2, *1/*3, *17/*2, and *17/*3	*2/*2, *2/*3, and *3/*3
Clopidogrel					
• CPIC	Use standard dose	Use standard dose	Use standard dose	Avoid clopidogrel. If cannot be avoided, triple the dose to 225 mg/day	Avoid clopidogrel
• DPWG	Use standard dose	Use standard dose	Use standard dose	Choose an alternative or double the dose to 150 mg/day	Avoid clopidogrel
PPIs					
• CPIC	Increase starting dose by 100%	Initiate standard dose. Consider increasing dose by 50%–100% for *Helicobacter pylori* infection and erosive esophagitis	Initiate standard dose. Consider increasing dose by 50%–100% for *Helicobacter pylori* infection and erosive esophagitis	Initiate standard dose. Consider 50% reduction in daily dose	Initiate standard dose. Consider 50% reduction in daily dose
• DPWG	Lansoprazole: use a 4-fold higher dose Omeprazole: use a 3-fold higher dose Pantoprazole: use a 5-fold higher dose	Initiate standard dose	Initiate standard dose	Lansoprazole: initiate standard dose Omeprazole and pantoprazole: an increase in the therapeutic effectiveness, without an increase in side effect	Lansoprazole: initiate standard dose Omeprazole and pantoprazole: an increase in the therapeutic effectiveness, without an increase in side effect
Voriconazole					
• CPIC	Choose an alternative agent	Choose an alternative agent	Initiate standard dose	Initiate standard dose	Choose an alternative agent
• DPWG	Use 50% of the standard dose and monitor the plasma concentration	Initiate standard dose	Initiate standard dose	Monitor the plasma concentration	Use an initial dose that is 1.5x higher and monitor the plasma concentration

### 2.4 Measures of treatment effect

The frequencies of the *CYP2C19* allele were presented as proportions and classified by country and race in a Table. In the absence of phenotype distribution details in the article, we used Hardy-Weinberg Equilibrium to estimate the phenotype distribution (Mayo, 2008). Single proportion meta-analyses of allele distribution were conducted using The R Project for Statistical Computing version 4.1.1 by inverse variance analysis. Furthermore, the efficacy and safety of drugs metabolized by *CYP2C19* were also subjected to meta-analysis, if feasible. Meta-analyses regarding clinical outcomes were conducted using Review Manager 5.4 by the Mantel-Haenszel method. All meta-analyses were conducted using a random effects model to account for potential variations, including genetic testing differences, racial differences, and discrepancies in outcome evaluation methods.

## 3 Results

**FIGURE 1:**
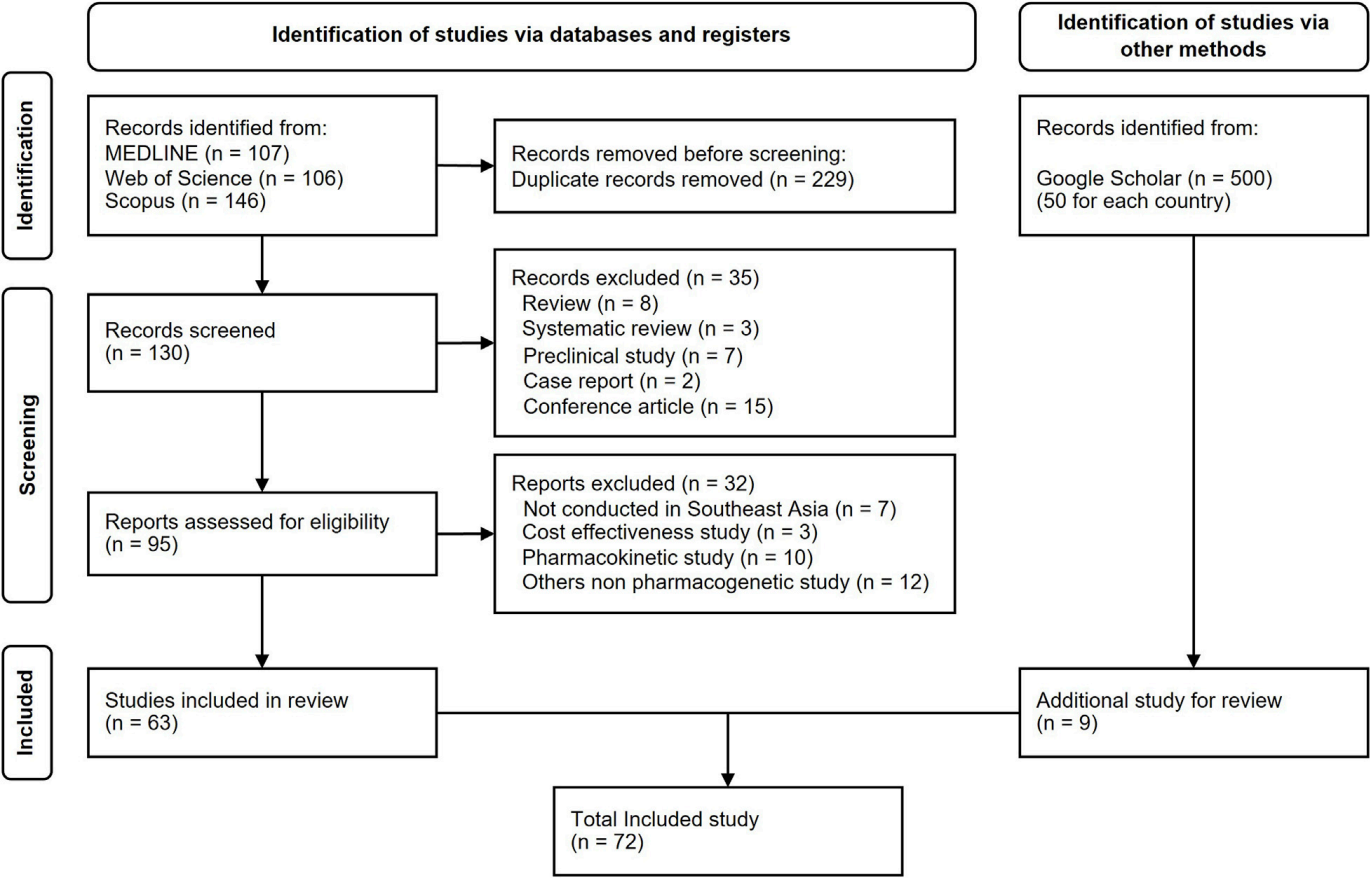
PRISMA flowchart.

Our systematic search produced 359 articles from databases, with the search strategies for each country and results displayed in Supplementary Table S2. After applying the inclusion and exclusion criteria, we identified 63 articles. This finding was supplemented by nine additional studies from Google Scholar, resulting in a total of 72 studies included in this meta-analysis. The PRISMA flowchart for this meta-analysis is illustrated in Figure 1.

Allele distribution studies in *CYP2C19* were predominantly conducted in Thailand (46.6%), followed by Indonesia (15.1%), with Singapore and Malaysia each representing 12.3% and 10.9%, respectively. We assessed and classified four medications linked to *CYP2C19* polymorphism according to the most recent CPIC guidelines, revealing that pharmacogenetic studies on clopidogrel, proton pump inhibitors (PPIs), voriconazole, and antidepressants have been performed 15, 12, 2, and 0 times, respectively. The full distribution of the included study for each country is presented in Supplementary Table S3. We also obtained data for additional drugs tested in relation to *CYP2C19*, including warfarin, tamoxifen, gliclazide, cyclophosphamide, and phenytoin, as detailed in Supplementary Table S4.

The risk of bias in the included studies is presented in Supplementary Table S5. The majority of bias risks came from a lack of clarification regarding confounding factors and their mitigation strategies. Thirteen studies (18.1%) exhibit a high risk of bias, whereas twenty-nine studies (40.3%) demonstrate a moderate risk of bias. We excluded three studies from the allele distribution analysis due to significant publication bias and inadequate data retrieval (Ram et al., 2019; Rochmawati et al., 2021; Hidayat et al., 2023), among the 13 studies identified with a high risk of bias.

### 3.1 *CYP2C19* variability in Southeast Asian populations

We obtained a comprehensive table of *CYP2C19* allele frequencies and phenotypes in the Southeast Asian population, categorized by country and ethnic groups (Supplementary Table S6). For the UM phenotype, most studies indicated less than 1% prevalence of Southeast Asian UM, with the highest prevalence recorded at 14.4% among Indian Singaporeans. The RM phenotype is also most prevalent among Indian Singaporeans, with a rate of 30.3%, while other races and countries exhibit prevalence rates below 10% (Lim et al., 2011; 2016). The highest prevalence of IM phenotype is 62.5% in Indonesia, although the prevalence varies among different races, ranging from 28.6% to 62.5%. The high prevalence of IM is similarly observed in other Southeast Asian nations, with rates typically ranging from 30% to 50%. The PM phenotype exhibits the highest prevalence in Indonesia, reaching up to 57.1% in a study of Papuan Indonesians (Miftahussurur et al., 2021).

The frequencies of the *2, *3, and *17 alleles in Chinese Singaporeans are 0.306, 0.059, and 0.009, respectively. In Malay and Indian Singaporeans, the proportions are 0.269, 0.052, 0.043 and 0.367, 0.009, 0.214, respectively. The allele distribution in Malaysia closely resembles that of Singapore’s Chinese, Malay, and Indian populations. East Malaysia also consists of Iban ethnic groups, with higher frequencies of *3 compared to the rest of Malaysia. Besides these ethnic groups, there are also indigenous people known as Orang Asli (Negritos, Senoi, and Proto-Malays). The Orang Asli exhibited a lower prevalence of *2 variance and a higher prevalence of *17 in comparison to the general Malaysian population. The Orang Asli population exhibits a high prevalence of the CYP2C19*35 loss-of-function allele, occurring at a frequency of 2.4% (Ang et al., 2016).

Thailand displays CYP2C19 *2, *3, and *17 alleles with frequencies of 0.279, 0.039, and 0.020, respectively, whereas Indonesia has rates of 0.248, 0.066, and 0.044, respectively. We observed significant heterogeneity in the studies of those alleles. We propose that this heterogeneity may arise from variations in polymorphism testing methodologies and the diverse racial backgrounds of the subjects.

In Vietnam, the frequencies of the CYP2C19 *2, *3, and *17 alleles are 0.293, 0.050, and 0.020, respectively. A study demonstrated that the prevalence of the Kinh *2 allele is lower than that in other ethnic groups in Vietnam (Vu et al., 2019). Several countries, such as Cambodia, Myanmar, and the Philippines, exhibit limited studies, revealing CYP2C19*2 prevalence rates of 0.275, 0.267, and 0.323, respectively. Table 2 outlined the distribution of alleles by country and ethnic group, while Supplementary Figure S1 illustrated the associated forest plots of allele distribution.

**TABLE 2:** Meta-analysis of single proportions in allele distribution by country and ethnicity.

No	Country – race – allele	Number of studies	Number of alleles studied	Proportion	95% confidence interval [lower bound; upper bound]	I^2^ heterogeneity (%)	References
1	Singapore						Sandanaraj et al. (2009), Lim et al. (2011), 2016; Chan et al. (2012), Brunham et al. (2014), Goh et al. (2017), Tan et al. (2020), Kothary et al. (2021), Soon et al. (2022)
	Chinese						
	*2	7	2008	0.3056	[0.2738; 0.3392]	54.4	
	*3	8	2,184	0.0587	[0.0495; 0.0695]	0.0	
	*17	5	1,060	0.0096	[0.0052; 0.0178]	0.0	
	Malay						
	*2	6	926	0.2687	[0.2390; 0.3007]	5.6	
	*3	7	1,100	0.0516	[0.0397; 0.0668]	0.0	
	*17	5	840	0.0431	[0.0237; 0.0771]	71.8	
	Indian						
	*2	6	1,008	0.3674	[0.3315; 0.4048]	47.3	
	*3	7	1,164	0.0089	[0.0048; 0.0164]	0.0	
	*17	5	918	0.2141	[0.1462; 0.3023]	89.2	
2	Malaysia						Yang et al. (2004), Mejin et al. (2013), Nasyuhana Sani et al. (2013), Yusoff, 2015; Ang et al. (2016), Tan et al. (2017), Zakaria et al. (2018), Ram et al. (2019)
	Chinese						
	*2	5	994	0.3028	[0.2654; 0.3431]	38.6	
	*3	4	750	0.0297	[0.0100; 0.0851]	85.5	
	*17	1	114	0.0263	-	-	
	Malay						
	*2	5	772	0.2184	[0.1906; 0.2490]	0.0	
	*3	4	652	0.0486	[0.0249; 0.0927]	65.7	
	*17	1	58	0.0172	-	-	
	Indian						
	*2	3	454	0.3154	[0.2742; 0.3597]	0.0	
	*3	3	454	0.0200	[0.0104; 0.0380]	0.0	
	Iban						
	*2	2	134	0.2177	[0.1526; 0.3007]	7.4	
	*3	1	48	0.1042	-	-	
	*17	1	48	0.0208	-	-	
3	Thailand						Yamada et al. (2001), Tassaneeyakul et al. (2002), 2006; Ngamjanyaporn et al. (2011), Manuyakorn et al. (2013), Prasertpetmanee et al. (2013), Sukasem et al. (2013), 2016; Chamnanphon et al., 2013, Srinarong et al. (2014), Tresukosol et al. (2014), Jainan and Vilaichone (2015), Nun-anan et al. (2015), Prapitpaiboon et al. (2015), Chuwongwattana et al. (2016), 2020; Jittikoon et al. (2016), Phiphatpatthamaamphan et al. (2016), Chotivitayatarakorn et al. (2017), Chunlertlith et al. (2017), Yampayon et al. (2017), Pussadhamma et al. (2018), Auttajaroon et al. (2019), Poonyam et al. (2019), Areesinpitak et al. (2020), Chamnanphon et al. (2020), Jirungda et al. (2020), Mauleekoonphairoj et al. (2020), Satapornpong et al. (2021), Sirivarasai et al. (2021), Sukprasong et al. (2021), Wankaew et al. (2022), John et al. (2024), Nakhonsri et al. (2024)
	*2	20	13,270	0.2789	[0.2582; 0.3005]	74.9	
	*3	17	12,146	0.0394	[0.0332; 0.0468]	72.2	
	*17	8	6,790	0.0203	[0.0122; 0.0337]	85.4	
4	Indonesia						Rusdiana et al. (2013), Ikawati et al. (2015), Rahmatini et al. (2018), Kothary et al. (2021), Miftahussurur et al. (2021), Rochmawati et al. (2021), Sukmawan et al. (2021), Tuba et al. (2021), Liem et al. (2022), Giantini et al. (2023), Hidayat et al. (2023)
	*2	6	1,512	0.2481	[0.1990; 0.3047]	82.6	
	*3	6	1,512	0.0662	[0.0380; 0.1128]	90.8	
	*17	3	464	0.0438	[0.0258; 0.0733]	54.8	
5	Vietnam						Yamada et al. (2001), Lee et al. (2007), Veiga et al. (2009), Vu et al. (2019), Hoang et al. (2022), Thuy et al. (2024)
	*2	6	2,234	0.2934	[0.2640; 0.3247]	60.5	
	*3	6	2,234	0.0506	[0.0288; 0.0874]	87.7	
	*17	2	1,162	0.0203	[0.0047; 0.0839]	91.1	
6	Cambodia						Bertrand et al. (2012), Hodel et al. (2013), Aumpan et al. (2020)
	*2	1	258	0.2751	-	-	
	*3	1	246	0.0285	-	-	
7	Myanmar						Win et al. (2016)
	*2	1	300	0.2667	-	-	
8	Philippines						Zumaraga et al. (2022)
	*2	1	254	0.3228	-	-	

### 3.2 *CYP2C19* variability and clopidogrel

Fifteen studies examining the variability of *CYP2C19* and clopidogrel in the Southeast Asian population. A physiology based-population pharmacokinetic (PBPK) model in the Malaysian population demonstrated that the plasma levels of the clopidogrel active metabolite (clopi-H4) in the PM phenotype were significantly lower than those in the EM phenotype (p < 0.001) (Zakaria et al., 2018).

**FIGURE 2:**
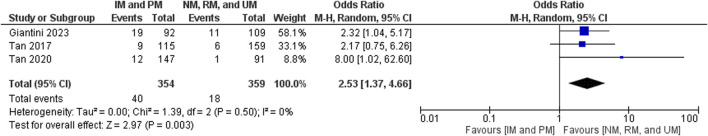
Forest plot comparing the *CYP2C19* intermediate metabolizer (IM) and poor metabolizer (PM) phenotypes to the normal metabolizer (NM), rapid metabolizer (RM), and ultra-rapid metabolizer (UM) phenotypes regarding clopidogrel major adverse cardiovascular events (MACE).

Four studies examined the correlation between *CYP2C19* polymorphism and major adverse cardiovascular events (MACE) (Mejin et al., 2013; Tan et al., 2017; 2020; Giantini et al., 2023). Our meta-analysis found that the risk of MACE in 12 months escalated by 2.53 times in individuals having the *CYP2C19* IM or PM phenotype (95% CI = 1.37 to 4.66, p = 0.003, I^2^ = 0%) (Table 3; Figure 2). A study within this meta-analysis demonstrated that MACE increased according to the category of individual metabolizer, with rates of 0%, 1.2%, 7.1%, and 11.4% for UM, EM, IM, and PM, respectively (Tan et al., 2020). Regarding mortality rate, a study found that three subjects experienced cardiovascular death, two had hospital readmission, but no significant differences were observed related to *CYP2C19* polymorphism (Mejin et al., 2013). Another study indicated that case mortality did not differ among various *CYP2C19* phenotypes (Pussadhamma et al., 2018).

**TABLE 3:** Summary of forest plot regarding the effectiveness of clopidogrel based on *CYP2C19* variability in Southeast Asian populations.

No.	Outcome or subgroup (PM, IM vs. NM, RM, UM)	Studies	Participants	Or [95%CI]	p	I^2^
1	MACE (major adverse cardiovascular events)	3	713	2.53 [1.37, 4.66]	0.003	0
2	Platelet aggregation	5	900	1.59 [1.12, 2.27]	0.01	24
	Subgroup by *CYP2C19* phenotype					
	• IM vs. NM, RM, UM	3	519	1.17 [0.76, 1.79]	0.47	0
	• PM vs. NM, RM, UM	3	371	4.32 [1.15, 16.15]	0.03	76
	Subgroup by platelet aggregation testing method					
	• light transmission aggregometry (LTA)	2	297	1.55 [0.96, 2.51]	0.07	0
	• Multiple electrode platelet aggregometry (MEA)	3	603	1.61 [0.84, 3.08]	0.15	62
3	Clopidogrel resistance	4	497	3.18 [1.64, 6.19]	0.0006	40
	Subgroup by *CYP2C19* phenotype					
	• IM vs. NM, RM, UM	2	241	4.65 [2.00, 10.77]	0.0003	0
	• PM vs. NM, RM, UM	2	150	13.39 [2.22, 80.80]	0.005	36
	Subgroup by clopidogrel aggregation testing method					
	• VerifyNow™	3	452	2.65 [1.39, 5.05]	0.003	30
	• INNOVANCE^®^ PFA P2Y	1	45	8.40 [1.94, 36.43]	0.005	-

Five studies analyzed clopidogrel effectiveness by measuring platelet aggregation after clopidogrel administration (Mejin et al., 2013; Sukasem et al., 2013; Tresukosol et al., 2014; Tan et al., 2017; Giantini et al., 2023). This meta-analysis indicates that individuals with *CYP2C19* phenotypes IM and PM have a 1.59-fold increased likelihood of being nonresponders according to platelet aggregation testing (95% CI = 1.12 to 2.27, p = 0.01, I^2^ = 24%). Subgroup analysis indicated that only the PM phenotype significantly elevated the nonresponder proportion by 4.32 times (95% CI = 1.15 to 16.15, p = 0.03, I^2^ = 76%), while the IM subgroup yielded non-significant results (Table 3; Supplementary Figure S11).

We identified potential heterogeneity resulting from various testing methodologies. Two studies evaluated only *2 genotype, neglecting *3 (Tan et al., 2017; Giantini et al., 2023). Three studies employed multiple electrode platelet aggregometry (MEA) (Mejin et al., 2013; Tresukosol et al., 2014; Tan et al., 2017), whereas the remaining two utilized the light transmission aggregometry (LTA) technique (Sukasem et al., 2013; Giantini et al., 2023). We conducted a subgroup analysis of the LTA and MEA procedures and observed that both methods tend to increase platelet aggregation by 1.55 and 1.61 times, respectively, but this finding did not achieve statistical significance (Supplementary Figure S12). A study examined the platelet reactivity index quantitatively, revealing that the IM phenotype exhibited an 11.6% greater platelet reactivity index than the EM phenotype (p = 0.001), whereas the PM phenotype demonstrated a 19.6% increase (p < 0.001) (Chan et al., 2012).

Four studies evaluated clopidogrel resistance associated with *CYP2C19* variability utilizing rapid platelet function tests (VerifyNow™ or INNOVANCE^®^ PFA P2Y) (Nasyuhana Sani et al., 2013; Jirungda et al., 2020; Tan et al., 2020; Sukmawan et al., 2021). The risk of clopidogrel resistance increased by 3.18 times in individuals with IM and PM phenotypes compared to those with NM, RM, and UM phenotypes (95% CI = 1.64 to 6.19, p = 0.0006, I^2^ = 40%). Subgroup analysis for IM and PM indicates that the PM phenotype exhibits a greater risk of clopidogrel resistance, with an odds ratio of 13.39 compared to 4.65 (Table 3; Supplementary Figure S13).

The heterogeneity among studies is likely due to the type of rapid platelet function tests employed and the definition of clopidogrel resistance as indicated by the P2Y12 reaction unit (PRU). One study used a PRU cutoff of 208, while another used 230 (Tan et al., 2020; Sukmawan et al., 2021). A study in this meta-analysis revealed that the incidence of clopidogrel resistance escalated in direct correlation with the variations of UM, EM, IM, and PM, with respective percentages of 0%, 6.3%, 20.5%, and 31.4% (Tan et al., 2020). One study quantitatively evaluated PRU across various phenotypes, revealing a progressive increase in PRU values of 157, 170, and 184 for EM, IM, and PM, respectively (Rahmatini et al., 2018). We conducted a subgroup analysis on rapid platelet function tests and discovered that VerifyNow™ (3 studies) significantly raised the odds ratio by 2.65-fold in identifying clopidogrel resistance in IM and PM populations, whereas INNOVANCE^®^ PFA P2Y (1 study) exhibited an odds ratio of 8.40 times (Table 3; Supplementary Figure S14). Nevertheless, because of the small number of studies, we were unable to draw conclusions regarding this discrepancy.

Two studies conducted in Indonesia evaluated the association between *CYP2C19* DNA methylation and clopidogrel resistance (Sukmawan et al., 2021; Giantini et al., 2023). Our meta-analysis revealed that DNA methylation levels below 50% were associated with a 2.37-fold increase (95% CI = 1.29 to 4.37, p = 0.006, I^2^ = 0%) in the risk of clopidogrel resistance compared to levels at or above 50% (Supplementary Figure S15).

### 3.3 *CYP2C19* variability and proton pump inhibitor

Seven studies conducted in Thailand, along with one each in Vietnam and Malaysia, evaluated the impact of *CYP2C19* variability on the eradication rate of *Helicobacter pylori* using various regimens and PPIs. Two studies utilized lansoprazole (Prasertpetmanee et al., 2013; Srinarong et al., 2014), three employed dexlansoprazole (Prapitpaiboon et al., 2015; Chotivitayatarakorn et al., 2017; Poonyam et al., 2019), and one study each involved omeprazole (Chunlertlith et al., 2017), esomeprazole (Thuy et al., 2024), and rabeprazole (Phiphatpatthamaamphan et al., 2016). The risk of treatment failure in individuals with NM and IM phenotypes tends to increase by 1.43 (95% CI = 0.62 to 3.28, p = 0.40) and 1.96 (95% CI = 0.85 to 4.55, p = 0.12) times, respectively, compared to PM individuals (Table 4; Supplementary Figure S16). A study in Malaysia indicated that the IM phenotype has a 1.5-fold higher eradication rate compared to the NM phenotype, whereas a combination of the IM and PM phenotypes exhibits a 1.49-fold increase (Ram et al., 2019). We conducted a subgroup analysis of the types of PPIs utilized, and none demonstrated statistical significance (Table 4; Supplementary Figure S17).

**TABLE 4:** Summary of forest plot regarding the effectiveness of proton pump inhibitors based on *CYP2C19* variability in Southeast Asian populations.

No.	Outcome or subgroup	Studies	Participants	Or [95%CI]	p	I^2^
1	NM, RM, and UM vs. PM	8	515	1.43 [0.62, 3.28]	0.40	0
	• Lansoprazole	2	95	1.06 [0.11, 10.15]	0.96	0
	• Dexlansoprazole	3	169	1.56 [0.37, 6.59]	0.55	0
	• Omeprazole	1	90	0.97 [0.19, 4.98]	0.97	-
	• Esomeprazole	1	111	7.48 [0.95, 58.84]	0.06	-
	• Rabeprazole	1	50	0.37 [0.03, 4.22]	0.81	-
2	IM vs. PM	8	495	1.96 [0.85, 4.55]	0.73	0
	• Lansoprazole	2	56	3.18 [0.14, 73.03]	0.47	-
	• Dexlansoprazole	3	167	1.41 [0.34, 5.82]	0.64	0
	• Omeprazole	1	94	2.75 [0.58, 13.11]	0.20	-
	• Esomeprazole	1	124	5.40 [0.68, 42.66]	0.11	-
	• Rabeprazole	1	54	0.45 [0.04, 4.90]	0.65	-

Two studies evaluated *CYP2C19* genetic polymorphism in patients with gastroduodenal disorders ultilizing PPIs (Supplementary Table S7) (Jainan and Vilaichone, 2015; Miftahussurur et al., 2021). In patients with gastritis, the prevalence of IM ranged from 40 to 53 percent, whereas the prevalence of PM ranged from 11 to 22 percent. The prevalence of IM in peptic ulcer ranged from 43 to 67 percent, whereas PM ranged from 9 to 17 percent. No significant difference was observed regarding the gastroscopy results and *CYP2C19* polymorphism.

### 3.4 *CYP2C19* variability and voriconazole

A study evaluating voriconazole plasma levels in 285 subjects indicated that plasma levels were significantly elevated in PM compared to EM (1.900 vs. 1.470 μg/mL, p = 0.039) and showed a tendency to be higher in IM compared to EM (1.860 vs. 1.470 μg/mL, p = 0.153) (Chuwongwattana et al., 2016). However, a follow-up study in the same institution indicated that only the children subgroup with the *1/*2 genotype exhibited elevated voriconazole plasma levels (1.130 vs 4.271 μg/mL, p = 0.038) (Chuwongwattana et al., 2020). This study also evaluated 146 adults and demonstrated no significance in voriconazole plasma levels across various *CYP2C19* genotypes.

## 4 Discussion

According to PharmVar, there are currently 39 variants in *CYP2C19*, with *1 representing the wild type with normal function, while the remaining variants (*2 to *39) are classified as defective alleles (Botton et al., 2021). Three primary variances exist: *2 and *3 represent loss-of-function alleles, while *17 represents an increased-function allele. The five *CYP2C19* phenotypes—UM, RM, NM, IM, and PM—are defined by the following haplotype combinations: UM is characterized by *17/*17, RM by *1/*17, NM by *1/*1, IM by *1/*2, *1/*3, *17/*2, and *17/*3, and PM by *2/*2, *2/*3, and *3/*3 (Caudle et al., 2017). The primary focus of this systematic review is these three variances due to their high prevalence in the population.

The most recent global meta-analysis indicated that the frequencies of CYP2C19*2, *3, and *17 polymorphisms in the East Asian population are 0.30, 0.07, and 0.02, respectively. In Central and South Asia, where most of the Indian population resides, the prevalence rates are 0.33, 0.01, and 0.15, respectively (Koopmans et al., 2021). Our systematic review indicated that the *CYP2C19* prevalence in Southeast Asia is comparable to that in East Asia, while the Indian population in Singapore or Malaysia exhibits a prevalence close to that of Central and South Asians. The Indian population in Singapore and Malaysia exhibits higher *17 polymorphism, which may suggest a greater incidence of PPI treatment failure for *H. pylori* infection in these populations relative to other areas of Southeast Asia. This form of ancestry influences pharmacogenetic variation and appears similar to a study evaluating *CYP2C19* polymorphism in British-South Asians, which revealed a high prevalence of RM and UM at 15% and 3%, respectively (Magavern et al., 2023).

Indonesia displays distinctive characteristics owing to Papua being associated with the Oceania population group, while the rest of Indonesia aligns with other Southeast Asian nations within the East Asia population group (Huddart et al., 2019). A previous review indicated that 50.1% of Melanesians, including those from Papua, are PM of *CYP2C19*, a rate higher than that observed in the rest of Southeast Asia (Helsby, 2016). This finding is corroborated by a study included in this meta-analysis, which indicated that the *CYP2C19* p.m. phenotype prevalence in Papuans is as high as 57.1%, whereas the rest of Indonesia exhibits a prevalence of 0%–25% (Miftahussurur et al., 2021). This might indicate a higher incidence of clopidogrel treatment failure among Papuans compared to other regions of Southeast Asia in the absence of pharmacogenetic testing.

A study in Thailand, where 97.5% of the population is Thai, revealed that the variance in CYP2C19 *2 across different areas was not statistically significant; however, the difference in *3 is likely negligible due to its low incidence (0.7%–3.2%) (Sukprasong et al., 2021). The geographical variance of *CYP2C19* across Southeast Asia may significantly impact the regulation of genotyping. The differences in genotype would result in variations in CYP2C19 phenotype. According to a global meta-analysis, the prevalence of the PM phenotype varies from less than 0.1% in Costa Rica to 31% in the Naik ethnic group in India. The UM phenotype varies from less than 0.1% in most regions of China to 38% in Pakistan (Koopmans et al., 2021). This difference in the CYP2C19 phenotype will influence the pharmacological response to drugs metabolized by CYP2C19. Nonetheless, these findings should be supplemented by research on drug utilization and *CYP2C19* polymorphism in Southeast Asian populations.

Clopidogrel is the most extensively studied in relation to *CYP2C19* in Southeast Asia. The active metabolite of clopidogrel, Clopi-H4, exhibits significant differences between the EM and PM phenotypes in Malay and Malaysian Chinese populations (Zakaria et al., 2018). This outcome aligns with a meta-analysis in a Caucasian population, which indicated that the area under the plasma concentration-time curve from administration to the last measurable concentration (AUC_0-t_) of clopidogrel active metabolites was 0.14 µM X hour lower in PM and IM compared to NM, RM, and UM (Holmes et al., 2011).

The absence of *CYP2C19* genotyping RCTs in Southeast Asia could reduce the validity of our meta-analysis, which relies solely on observational studies. Consequently, our meta-analysis could not determine the efficacy of guided genotyping in *CYP2C19* within Southeast Asian populations. However, our meta-analysis indicated that the Southeast Asian population demonstrates a high prevalence of IM and PM, which is significantly associated with increased MACE, platelet aggregation, and clopidogrel resistance, suggesting that genotyping may be more beneficial compared to European populations.

Our subgroup analysis comparing LTA and MEA techniques for detecting platelet aggregation revealed no significant differences, with ORs of 1.55 and 1.61, respectively. While LTA has established itself as the gold standard for assessing platelet aggregation, MEA offers advantages in clinical contexts due to its requirement for smaller blood volumes and less sample manipulation (Sun et al., 2019). Currently, no research elucidates the relationship between LTA and MEA in clopidogrel users, whereas such studies exist for ticagrelor and prasugrel users. The study demonstrated a significant correlation between LTA and MEA in prasugrel-treated individuals, but not in those treated with ticagrelor (Wadowski et al., 2021). Owing to the limited sample size, we were unable to ascertain conclusions regarding the disparity between these two approaches. In the subgroup analysis of VerifyNow™ and INNOVANCE^®^, conclusions could not also be drawn due to the limited sample size; nevertheless, another study indicated that the sensitivity of INNOVANCE^®^ is comparable to that of VerifyNow™ (Jang et al., 2012).

Our statement regarding the usefulness of *CYP2C19* genotyping for clopidogrel in Southeast Asia is supported by two cost-effectiveness analyses conducted in Singapore. Both studies demonstrated that *CYP2C19* genotyping is cost-effective for acute coronary syndrome and ischemic stroke, with incremental cost-effectiveness ratios (ICER) of SGD 88,991 and 33,839, respectively (Narasimhalu et al., 2020; Kim et al., 2021). Given that the ICER of *CYP2C19* genetic testing is lower than Singapore’s gross domestic product (GDP) *per capita*, it should be advanced into guidance for Singapore, as it is likely to be cost-effective in that context. This aligns with the new NICE guidance that recommends the use of *CYP2C19* to evaluate clopidogrel appropriateness for patients with ischemic stroke or transient ischemic attack (NICE, 2024). However, this result should be interpreted cautiously, as Singapore, Malaysia, and Brunei are the sole high-income countries (HICs) in Southeast Asia. Low-middle income countries (LMICs) have lower GDP *per capita*, which decreases the willingness to pay (WTP) and sets lower cost-effectiveness thresholds in comparison to HICs (Hardi and Barinda, 2024). Yet, no cost-effectiveness analyses have been conducted on *CYP2C19* utilization in Southeast Asian countries, except Singapore.

Besides differences in the *CYP2C19* genotype, gene expression variations can also be modulated through epigenetic mechanisms, such as the methylation process. While two studies reported an association between CYP2C19 hypomethylation and a 2.37-fold increase in clopidogrel resistance, the mechanistic basis of this result remains unknown. Notably, CYP2C19 CpG islands are located in intronic regions (introns one and 5), where methylation may not directly repress transcription (Burns et al., 2018). Methylation alterations in non-promoter regions, such as introns, may not substantially influence transcriptional activity and signify epigenetic signatures without functional implications (Glass et al., 2017). In addition, an *in vitro* study demonstrated that inhibiting the *CYP2C19* CpG islands with 5-aza-2′-deoxycytidine (5azaDC) resulted in upregulation of *CYP2C19* (Burns et al., 2018). Additional study incorporating methylation, expression, and phenotypic data is crucial to determine if CYP2C19 methylation status functionally influences clopidogrel resistance.

Drug interaction is also a significant factor in addition to *CYP2C19* polymorphism. The capacity of specific PPIs, particularly omeprazole and esomeprazole, to function as inhibitors of *CYP2C19* may additionally influence clopidogrel metabolism (El Rouby et al., 2018). A retrospective study in Singapore demonstrated that the co-prescription of clopidogrel and omeprazole elevates the risk of subsequent myocardial infarction by 2.03 times (Muthiah et al., 2021), signifying an increase in clopidogrel treatment failure. PPI metabolism is also affected by *CYP2C19* polymorphism, as explained in the subsequent paragraph.

Our meta-analysis indicated that the risk of PPI treatment failure in *H. pylori* eradication tends to elevate by 1.43 times in NM, RM, and UM compared to PM, and by 1.96 times in IM compared to PM. Nonetheless, our findings must be interpreted with caution due to three studies exhibiting zero events in PM, which may induce bias. The variation in PPI types and study designs, whether cohort or randomized controlled trials, might also introduce bias. We conducted a subgroup analysis of each type of PPI and found no significant results. This may be attributed to a limited sample size and numerous studies with zero events. Nonetheless, we discovered that the rabeprazole subgroup exhibits the lowest odds ratio compared to other PPIs, confirming rabeprazole’s limited metabolism by *CYP2C19* and indicating that dose adjustment is unnecessary in *CYP2C19* polymorphism, as advised by CPIC (Lim et al., 2005; Lima et al., 2021).

Our meta-analysis aligns with other meta-analyses indicating that *CYP2C19* polymorphism influences the cure rates of *H. pylori* (Ghazvini et al., 2021; Zhao et al., 2022). Nevertheless, a meta-analysis indicated that pantoprazole is less reliant on *CYP2C19* genotype (Zhao et al., 2022), a conclusion that our meta-analysis could not corroborate due to no study in Southeast Asia evaluating the use of pantoprazole for *H. pylori* eradication. A separate meta-analysis assessing only Asian populations indicated that *CYP2C19* polymorphism could affect *H. pylori* eradication rates only in China and Japan (Fu et al., 2021), whereas our meta-analysis also demonstrated its influence in Southeast Asian populations.

CPIC guidelines stated that patients with *CYP2C19* UM phenotype should have their initial daily dose increased by 100%, whereas NM, IM, and PM might consider a 50% reduction in daily dose for chronic therapy exceeding 12 weeks (Lima et al., 2021). Our meta-analysis demonstrated this propensity for treatment failure in NM, RM, and UM. In addition, we also investigated the potential for heightened *H. pylori* treatment failure in IM relative to PM within the Southeast Asian population. Nevertheless, additional research with a larger sample and consistent regimen should be conducted before a conclusion can be drawn.

Regarding *CYP2C19* variability and voriconazole plasma levels, our meta-analysis only found two studies in Southeast Asia. Hence, we were unable to draw conclusions regarding its implementation in the region. The CPIC guidelines indicated that alternative antifungals should be administered to individuals with *CYP2C19* UM, RM, and PM phenotypes (Moriyama et al., 2017). This guideline is supported with two global meta-analyses indicating that voriconazole plasma concentrations were markedly elevated in PM and IM compared to NM (Lee et al., 2021; Zhang et al., 2022). Nevertheless, regarding effectiveness and safety, one meta-analysis indicated that PM individuals have a 2.18-fold increased risk of overall adverse effects and a 1.6-fold increased risk of hepatotoxicity (Zhang et al., 2021), whereas another meta-analysis found no significant effects (Lee et al., 2021). Consequently, the application of *CYP2C19* genotyping in Southeast Asia requires further investigation prior to its routine implementation, particularly in terms of effectiveness and safety.

Many of our findings in this meta-analysis originate from Thailand (46.6%), while Cambodia, Myanmar, and the Philippines exhibit a limited number of studies. This could reduce the generalizability of this meta-analysis. Therefore, *CYP2C19* studies are strongly recommended in less researched countries. This meta-analysis indicates that the *CYP2C19* allele distribution in Southeast Asians is comparable to that of East Asians, except for Indians and Papuans. The *CYP2C19* polymorphism in Southeast Asia may result in therapeutic failure with clopidogrel and proton pump inhibitors, whereas voriconazole requires further investigation.

Upon the completion of additional research on *CYP2C19* in Southeast Asian populations, we can advance towards the integration of these studies into clinical practice. The latest paper by the Southeast Asian Pharmacogenomics Research Network (SEAPharm) revealed some limitations in the use of pharmacogenetic procedures, stating that in 2018, Singapore had just six clinical geneticists, Thailand had 22, and Malaysia had between 2 and 20 (Chong et al., 2018). Additionally, SEAPharm has established a recommendation for both the short and long future. In the short term, it is essential to enhance awareness among society and policymakers, as well as to improve educational programs. In the long term, it is crucial to promote a national precision medicine initiative and associated legislation, underpinned by sustainable public and commercial funding (Chong et al., 2018).

SEAPharm’s short-term plans demonstrate an acceptable degree of awareness among the public, policymakers, and patients, as evidenced by multiple studies indicating success. A Malaysian poll involving 221 healthcare providers and 200 patients or family members indicated a high intention (5.39 out of 7.00) to implement pharmacogenetic testing in their practice and for personal use, respectively (Mustapa et al., 2020). A subsequent poll in Malaysia revealed that 80% of community pharmacists desire to incorporate precision medicine into their daily practice, despite 61% lacking prior exposure to pharmacogenomics during their pharmacy education in Malaysia (Naimat et al., 2022). In addition, a survey of 150 patients in Singapore following pharmacogenetic testing revealed that 70% reported feelings of pleasure and happiness upon obtaining their test results, along with increased confidence in adhering to their recommended medicine (Ng et al., 2025).

Two Southeast Asia countries have developed their specific roadmaps, including Thailand’s 20-year genomic roadmap (https://genomicsthailand.com/Genomic/strategy) and Singapore’s 2030 strategic plan (https://www.a-star.edu.sg/docs/librariesprovider11/gis-pdf/fa_gis_strategic_plan_content.pdf). Thailand intends to create 10,000 individual human genome databases, implement genetic testing services utilizing next-generation sequencing technology, develop clinical guidelines, build policy and law, and facilitate cross-platform study of diverse genomic information. Singapore intends to create polygenic risk scores, incorporate pharmacogenetic variations into electronic health records (EHR) and wearable devices, investigate novel genes in pharmacogenetics, and integrate germline and somatic mutations using high-throughput functional assays. A multidisciplinary expert panel study in Malaysia identified three major barriers to the implementation of pharmacogenetics in clinical practice: the absence of a national policy or guideline, insufficient reimbursement for testing, and an underdeveloped health system for integrating pharmacogenetic data (Omran et al., 2024). As for now, the implementation policy and guideline for *CYP2C19* have not occurred in Southeast Asia. The most well-known implementation policies of pharmacogenetics are those of the Singapore Ministry of Health and Thailand’s public health regulations, which mandate HLA-B*15:02 allele genotyping for all patients prior to carbamazepine administration (Sukasem et al., 2021; Smith et al., 2024). Our meta-analysis indicated that CYP2C19 testing in patients administered clopidogrel deserves consideration for inclusion in policy or pharmacogenetic testing algorithms.

To enhance its integration into clinical practice, reimbursement for *CYP2C19* pharmacogenetic testing should be established. This has been implemented with another gene. For example, under the Thailand Universal Coverage Scheme (UCS), which covers 75% of the Thailand population, a reimbursement of $28 is provided for each HLA-B*15:02 allele genotyping (Tangcharoensathien et al., 2020; Sukasem et al., 2021). The average coverage for *CYP2C19* is $100 in the United States (US), according to Medicare and commercial insurance data (Anderson et al., 2020). This value may serve as a reference for establishing payment in Southeast Asia, where *CYP2C19* reimbursement has yet to be implemented.

Communicating the data to the clinician upon their availability is a vital component of pharmacogenetic practice. Incorporating pharmacogenetic findings into hospital electronic health records (EHR) is essential. However, in two hospitals in Thailand, pharmacogenetic test findings were not integrated into their EHR or drug prescription systems (Sukasem et al., 2021). Incorporating pharmacogenetic findings into EHR is challenging, as evidenced by a US hospital that established a system over a 4-year duration. Initially, the project started with the implementation of clopidogrel clinical decision systems (Peterson et al., 2013). This strategy can be replicated, as our meta-analysis identified several studies that investigated clopidogrel and *CYP2C19*. A different approach to utilizing pharmacogenetic ID cards has achieved significant success in Thailand. The primary concept is that patients may carry it and show it to physicians at any medical facility (Sukasem et al., 2021).

Despite numerous genomic initiatives across Southeast Asian nations, including the Genomics Thailand Initiative (GTI), the Indonesian Biomedical and Genome Science Initiative (BGSI), and the Singapore National Precision Medicine Initiative, Southeast Asian countries still lack adequate facilities for pharmacogenetic testing. For instance, the median turnaround time for pharmacogenetic testing (without EHR entry) was 3 days in Thailand and 5 days in Singapore (Sukasem et al., 2021; Ng et al., 2025). Approximately 7% of the sample in the Singapore study experienced delays attributable to operational challenges and technical constraints (Ng et al., 2025). In comparison, the turnaround time from blood draw to genotyping result entry in the EHR in the US is merely 3.5 days (Weitzel et al., 2014).

This meta-analysis exhibited several variabilities, including variations in genotyping methodologies, such as single-gene PCR and whole genome sequencing, as well as discrepancies in outcome assessment for each investigated drug. Consequently, the standardized research technique may be more effectively recommended to mitigate this type of heterogeneity. However, the results of this study can illustrate how the Southeast Asian genotype profile compares to that of neighboring regions, suggesting that pharmacogenetic practices may be more effectively adapted from Southeast Asian neighbors, such as East Asia, rather than from Europe or the United States.

## Data Availability

The original contributions presented in the study are included in the article/Supplementary Material, further inquiries can be directed to the corresponding author.
