# Supplementary material for: CYP2C19 variability and clinical outcomes of clopidogrel, proton pump inhibitors, and voriconazole in Southeast Asia: a systematic review and meta-analysis
Source: Front Pharmacol. 2025 Jun 19;16:1572886. doi: 10.3389/fphar.2025.1572886 (PMC12223402; doi:10.3389/fphar.2025.1572886)
Supplement: Supplementary file 1 [file DataSheet1.docx]

Supplementary Material: CYP2C19 Variability and Clinical Outcomes of Clopidogrel, Proton Pump Inhibitors, and Voriconazole in Southeast Asia: A Meta-Analysis

Contents

[Supplementary Table 1. Search strategy (performed on Oct 4, 2024) 3](#_Toc192103349)

[Supplementary Table 2. Search result for each country 4](#_Toc192103350)

[Supplementary Table 3. Distribution of included studies by country regarding allele distribution and the total number of each drug studied for which CYP2C19 guidance has been published by the Clinical Pharmacogenetics Implementation Consortium (CPIC). 5](#_Toc192103351)

[Supplementary Table 4. Pharmacogenetic studies which include clinical effectiveness and safety of drugs metabolized by CYP2C19 6](#_Toc192103352)

[Supplementary Table 5. Risk of bias among included study 14](#_Toc192103353)

[Supplementary Table 6. Detailed description on CYP2C19 allele frequency and phenotype in Southeast Asian population 17](#_Toc192103354)

[Supplementary Table 7. CYP2C19 polymorphism and clinical outcomes based on endoscopy 21](#_Toc192103355)

[Supplementary Figure 1. Meta-analysis of allele frequencies in Chinese Singaporeans: (a) for *2 alleles; (b) for *3 alleles; (c) for *17 alleles. 22](#_Toc192103356)

[Supplementary Figure 2. Meta-analysis of allele frequencies in Malay Singaporeans: (a) for *2 alleles; (b) for *3 alleles; (c) for *17 alleles. 23](#_Toc192103357)

[Supplementary Figure 3. Meta-analysis of allele frequencies in Indian Singaporeans: (a) for *2 alleles; (b) for *3 alleles; (c) for *17 alleles. 24](#_Toc192103358)

[Supplementary Figure 4. Meta-analysis of allele frequencies in Chinese Malaysians: (a) for *2 alleles; (b) for *3 alleles. 25](#_Toc192103359)

[Supplementary Figure 5. Meta-analysis of allele frequencies in Malay Malaysians: (a) for *2 alleles; (b) for *3 alleles. 26](#_Toc192103360)

[Supplementary Figure 6. Meta-analysis of allele frequencies in Indian Malaysians: (a) for *2 alleles; (b) for *3 alleles. 27](#_Toc192103361)

[Supplementary Figure 7. Meta-analysis of allele frequencies in Iban Malaysians for *2 alleles 28](#_Toc192103362)

[Supplementary Figure 8. Meta-analysis of allele frequencies in Thailand: (a) for *2 alleles; (b) for *3 alleles; (c) for *17 alleles 29](#_Toc192103363)

[Supplementary Figure 9. Meta-analysis of allele frequencies in Indonesia: (a) for *2 alleles; (b) for *3 alleles; (c) for *17 alleles 30](#_Toc192103364)

[Supplementary Figure 10. Meta-analysis of allele frequencies in Vietnam: (a) for *2 alleles; (b) for *3 alleles; (c) for *17 alleles 31](#_Toc192103365)

[Supplementary Figure 11. (a) Forest plot comparing the CYP2C19 intermediate metabolizer (IM) and poor metabolizer (PM) phenotypes to the normal metabolizer (NM), rapid metabolizer (RM), and ultra-rapid metabolizer (UM) phenotypes regarding platelet aggregation. (b) Subgroup analysis of IM. (c) Subgroup analysis of PM. 32](#_Toc192103366)

[Supplementary Figure 12. Subgroup analysis of platelet aggregation analysis according to its methodologies. (a) Subgroup analysis of light transmittion aggregation (LTA) method. (b) Subgroup analysis of multiple electrode platelet aggregometry (MEA) method. 33](#_Toc192103367)

[Supplementary Figure 13. (a) Forest plot comparing the CYP2C19 intermediate metabolizer (IM) and poor metabolizer (PM) phenotypes to the normal metabolizer (NM), rapid metabolizer (RM), and ultra-rapid metabolizer (UM) phenotypes regarding clopidogrel resistance. (b) Subgroup analysis of IM. (c) Subgroup analysis of PM. 34](#_Toc192103368)

[Supplementary Figure 14. Subgroup analysis of clopidogrel resistance based on VerifyNow^TM^ method 35](#_Toc192103369)

[Supplementary Figure 15. Forest plot comparing the CYP2C19 DNA methylation <50% to >=50% regarding clopidogrel resistance. 36](#_Toc192103370)

[Supplementary Figure 16. Forest plot comparing CYP2C19 phenotypes of (a) normal metabolizer (NM), rapid metabolizer (RM), and ultrarapid metabolizer (UM) versus poor metabolizer (PM) phenotype, and (b) intermediate metabolizer (IM) versus PM phenotype in relation to treatment failure in *Helicobacter pylori* infection on proton pump inhibitor treatment. 37](#_Toc192103371)

[Supplementary Figure 17. Forest plot comparing CYP2C19 phenotypes of (a) normal metabolizer (NM), rapid metabolizer (RM), and ultrarapid metabolizer (UM) versus poor metabolizer (PM) phenotype, and (b) intermediate metabolizer (IM) versus PM phenotype in relation to treatment failure in *Helicobacter pylori* infection on lansoprazole treatment. (c) NM, RM, UM versus PM phenotype, (d) IM versus PM phenotype on dexlansoprazole treatment 38](#_Toc192103372)

# Supplementary Table 1. Search strategy (performed on Oct 4, 2024)

| **Database** | **Keywords** |
| --- | --- |
| Pubmed | (“country keyword”[Title/Abstract]) AND (cyp2c19[Title/Abstract]) |
| Scopus | ( TITLE-ABS-KEY ( “country keyword” ) AND TITLE-ABS-KEY ( cyp2c19 ) ) |
| Web of Science | ((TI=(“country keyword”)) OR AB=(“country keyword”) AND (TI=(CYP2C19)) OR AB=(CYP2C19)) |

# Supplementary Table 2. Search result for each country

| **Country** | **Country Keyword** | **Pubmed (Medline)** | **Scopus** | **Web of Science** |
| --- | --- | --- | --- | --- |
| Brunei | Brunei | 0 | 0 | 0 |
| Cambodia | Cambodia* OR Khmer | 5 | 7 | 4 |
| Indonesia | Indonesia* | 5 | 13 | 6 |
| Lao PDR | Lao* | 0 | 1 | 1 |
| Malaysia | Malay* | 21 | 30 | 19 |
| Myanmar | Myanmar OR Burm* | 4 | 7 | 3 |
| Philippines | Philippines OR Filipino* | 4 | 7 | 5 |
| Singapore | Singapore* OR (Malay* AND Indian* AND Chinese) | 16 | 15 | 13 |
| Thailand | Thai* | 40 | 50 | 43 |
| Vietnam | Vietnam* OR Kinh | 12 | 16 | 12 |
| **Total** | | **107** | **146** | **106** |

# Supplementary Table 3. Distribution of included studies by country regarding allele distribution and the total number of each drug studied for which CYP2C19 guidance has been published by the Clinical Pharmacogenetics Implementation Consortium (CPIC).

| **Country** | **Allele distribution** | **Clopidogrel** | **PPI** | **Voriconazole** |
| --- | --- | --- | --- | --- |
| Brunei | 0 | 0 | 0 | 0 |
| Cambodia | 3 | 0 | 1 | 0 |
| Indonesia | 11 | 5 | 1 | 0 |
| Lao PDR | 0 | 0 | 0 | 0 |
| Malaysia | 8 | 4 | 1 | 0 |
| Myanmar | 1 | 0 | 0 | 0 |
| Philippines | 1 | 0 | 0 | 0 |
| Singapore | 9 | 2 | 0 | 0 |
| Thailand | 34 | 4 | 8 | 2 |
| Vietnam | 6 | 0 | 1 | 0 |
| **Total** | **73** | **15** | **12** | **2** |

Note: One study evaluated the allele distribution in two countries, Indonesia and Singapore, yielding a total of 73 findings from 72 included studies.

Supplementary Table 4. Pharmacogenetic studies which include clinical effectiveness and safety of drugs metabolized by CYP2C19

| No. | Drug Class | Country | Dosage | Type of Study | Total subject and subject characteristic | Result | Study |
| --- | --- | --- | --- | --- | --- | --- | --- |
| 1. | Proton Pump Inhibitor | Thailand | Lansoprazole 60 mg BID | Cohort prospective | 64 patients with *H. pylori* infection | Effectiveness: *H. pylori* eradication rate  NM: responder 34, non-responder 2  IM: responder 19, non-responder 0  PM: responder 9, non-responder 0 | Prasertpetmanee  (2013) |
|  |  |  | Lansoprazole 30 mg BID | RCT | 98 patients with *H. pylori* infection | Effectiveness: *H. pylori* eradication rate  NM: responder 39, non-responder 1  IM: responder 16, non-responder 2  PM: responder 10, non-responder 0 | Srinarong  (2014) |
|  |  |  | Dexlansoprazole 60 mg BID | RCT | 98 patients with *H. pylori* infection | Effectiveness: *H. pylori* eradication rate  NM: responder 49, non-responder 4  IM: responder 31, non-responder 3  PM: responder 11, non-responder 0 | Prapitpaiboon  (2015) |
|  |  |  | Dexlansoprazole 60 mg BID | RCT | 103 patients with *H. pylori* infection | Effectiveness: *H. pylori* eradication rate  NM: responder 42, non-responder 2  IM: responder 45, non-responder 3  PM: responder 10, non-responder 1 | Chotivitayatarakorn (2017) |
|  |  |  | Dexlansoprazole 60 mg BID | RCT | 100 patients with *H. pylori* infection | Effectiveness: *H. pylori* eradication rate  NM: responder 29, non-responder 8  IM: responder 43, non-responder 7  PM: responder 12, non-responder 1 | Poonyam (2019) |
|  |  |  | Omeprazole 20 mg BID | RCT | 85 patients with *H. pylori* infection | Effectiveness: *H. pylori* eradication rate  NM: responder 28, non-responder 6  IM: responder 27, non-responder 13  PM: responder 10, non-responder 1 | Chunlertlith  (2017) |
|  |  |  | Omeprazole 20 mg TID | RCT | 85 patients with *H. pylori* infection | Effectiveness: *H. pylori* eradication rate  NM: responder 37, non-responder 3  IM: responder 29, non-responder 9  PM: responder 4, non-responder 1 |  |
|  |  |  | Rabeprazole 20 mg BID | RCT | 98 patients with *H. pylori* infection | Effectiveness: *H. pylori* eradication rate  NM: responder 42, non-responder 2  IM: responder 44, non-responder 4  PM: responder 5, non-responder 1 | Phiphatpatthamaamphan (2016) |
|  |  | Vietnam | Esomeprazole 20-40 mg BID | Cohort prospective | 207 children with *H. pylori* infection | Effectiveness: *H. pylori* eradication rate  NM: responder 65, non-responder 18  IM: responder 80, non-responder 16  PM: responder 27, non-responder 1 | Thuy  (2024) |
| 2. | Antiplatelet | Malaysia | Clopidogrel 75 mg | PBPK modelling | Database from Malaysia government | Effectiveness and safety: clopidrogrel metabolite (clopi-H4) plasma level   - Clopi H-4 plasma level in PM significantly lower compared to EM (p < 0.001) - High-dose strategy (600 mg followed by 150 mg) increase 50% subject in attaining minimum clopi-H4 plasma concentration for a therapeutic effect | Zakaria (2018) |
|  |  |  | Clopidogrel 300 mg, assessed after 4 h of administration | Pharmacokinetic study in healthy person | 45 healthy subject | Effectiveness: Platelet reactivity unit (PRU); mean (SD); only assessing *2  EM: 147.7 + 87.2  IM: 232.5 + 81.4  PM: 291.0 + 62.1 | Sani (2013) |
|  |  |  | Clopidogrel 75 mg for at least 4 days | Prospective cohort | 118 subject who being listed for PCI | Effectiveness: ADP > 400, multiple electrode aggregometry  UM: responder 1, nonresponder 0  RM: responder 7, nonresponder 0  NM: responder 35, nonresponder 8  IM and PM: responder 52, nonresponder 15  No bleeding was observed  No significant difference in MACE was observed | Mejin (2013) |
|  |  |  | Clopidogrel 75 mg daily | Cohort prospective | 274 patients with after PCI | Effectiveness: 1 year MACE; only assessing *2  EM: MACE 6, no MACE 153  IM and PM: MACE 9, no MACE 106  OR 2.17 (95% CI: 0.75 – 6.26)  Effectiveness: ADP-induced aggregation; only assessing *2  EM: 397.4 ± 223.3 AU*min  IM and PM: 435.0 ± 206.0 AU*min  (p = 0.152)  EM: responder 127, nonresponder 32  IM: responder 78, nonresponder 17  PM: responder 14, nonresponder 6  Bleeding: 3 major bleeding (1.1%) and 19 minimal bleeding (6.8%) | Tan (2017) |
|  |  | Singapore | Clopidogrel 300 mg, followed by 75 mg daily for 5-7 days | Cross sectional | 89 patients with clopidogrel treatment | Effectiveness: platelet reactivity index (%)   - IM phenotype has 11.6% (95% CI: 5.32–18.6%; p = 0.001) higher platelet reactivity index compared to RM - PM phenotype has 19.6% (95% CI: 9.3–29.9%; p < 0.001) higher platelet reactivity index compared to RM | Chan (2012) |
|  |  |  | Clopidogrel 75 mg daily | Cohort prospective | 199 patients with after PCI | Effectiveness: clopidogrel resistance  UM: resistance 0, nonresistance 12  EM: resistance 5, nonresistance 74  IM: resistance 23, nonresistance 89  PM: resistance 11, nonresistance 24  Effectiveness: 1 year MACE  UM: MACE 0, no MACE 12  EM: MACE 1, no MACE 78  IM: MACE 8, no MACE 104  OR 6.0 (95% CI: 0.7‐49.0, p = 0.094)  PM: MACE 4, no MACE 31  OR 10.1 (95% CI: 1.1‐93.6, p = 0.042)  The incidence rates of MACE for CYP2C19 UM, EM, IM and PM phenotypes were 0%, 1.3%, 7.1% and 11.4% at 12 months post‐index PCI  Safety: bleeding  UM: bleeding 1 (8.3%), no bleeding 11  EM: bleeding 9 (11.4%), no bleeding 70  IM: bleeding 18 (16.1%), no bleeding 94  PM: bleeding 2 (5.7%), no bleeding 33 | Tan (2020) |
|  |  | Thailand | Clopidogrel 75 mg for at least 14 days | Cohort retrospective | 96 CAD patients | Effectiveness: platelet response; light transmission aggregometry  UM: responder 2, non-responder 0  EM: responder 32, non-responder 9  IM: responder 26, non-responder 7  PM: responder 11, non-responder 9 | Sukasem (2013) |
|  |  |  | Clopidogrel 75 mg for at least 14 days | Cohort prospective | 211 CAD patients | Effectiveness: platelet aggregation; multiple electrode aggregometry  NM: responder 82, non-responder 24  IM: responder 55, non-responder 27  OR: 1.677 (95%CI: 0.877 to 3.204, p=0.117)  PM: responder 4, non-responder 19  OR: 16.229 (95%CI: 5.036 to 52.304, p<0.0001) | Tresukosol  (2014) |
|  |  |  | Clopidogrel 75 mg for at least one week | Cohort prospective | 67 Recurrent Myocardial Infarction after Coronary Stenting | Effectiveness: Platelet reactivity unit (PRU); median (range)  EM: 213 (90 to 368)  IM: 237 (8 to 357), p = 0.98  PM: 234 (62 to 353), p = 0.67  Effectiveness: 5-year survival  EM: 68% (46 to 83%)  IM + PM: 67% (50 to 79%)  Effectiveness: 3-year survival  IM: 68% (49 to 82%)  PM: 76% (33 to 95%) | Pussadhamma (2018) |
|  |  |  | Clopidogrel 75 mg for at least one week | Cohort prospective | 102 ACS subjects prior PCI | Effectiveness: platelet aggregation; INNOVANCE-PFA P2Y test (PFA 200)  NM: responder 25, non-responder 29  IM+PM: responder 9, non-responder 39 | Jirangda (2019) |
|  |  | Indonesia | Clopidogrel 300 mg, 48 h after loading dose | Cross-sectional | 30 ACS subjects | Effectiveness: Platelet reactivity unit (PRU); mean + SD  EM: 157 + 66  IM: 170 + 79  PM: 184 + 91 | Rahmatini (2018) |
|  |  |  | Clopidogrel | Cross-sectional | 53 patients with coronary heart disease | Effectiveness and safety: platelet aggregation (only *3)  NM: hypoaggregation 35, normal aggregation 5, hyper aggregation 0  IM: hypoaggregation 2, normal aggregation 0, hyper aggregation 0  PM: hypoaggregation 10, normal aggregation 1, hyper aggregation 0 | Rochmawati (2021) |
|  |  |  | Clopidogrel 600 mg loading dose | Cross-sectional | 122 CAD patients pre-PCI | Effectiveness: clopidogrel resistance  EM: resistance 14, nonresistance 62  IM and PM: resistance 13, nonresistance 33  OR: 1.7 (95% CI: 1.1 – 7.4)  Effectiveness: TIMI flow after PCI  EM: TIMI flow 3: 55, TIMI flow <3: 21  IM and PM: TIMI flow 3: 30, TIMI flow <3: 16  OR: 1.3 (95% CI: 0.6 – 3.0) | Sukmawan (2021) |
|  |  |  | Clopidogrel | Cross-sectional | 112 ischemic stroke patients with CYP2C19*17 | No difference risk of bleeding between rapid, intermediate, and poor metabolizer (p = 0.44)  RM: bleeding 9, no bleeding 10  IM: bleeding 18, no bleeding 35  PM: bleeding 18, no bleeding 22 | Hidayat (2023) |
|  |  |  | Clopidogrel 75 mg daily | Prospective Cohort | 201 ACS post-PCI | Effectiveness: clopidogrel resistance (LTA); only assessing *2  EM: resistance 49, nonresistance 60  IM and PM: resistance 51, nonresistance 41  Effectiveness: MACE  EM: MACE 11, no MACE 98  IM and PM: MACE 19, no MACE 73 | Giantini (2023) |
| 3 | Anticoagulant | Singapore | Warfarin | Cohort prospective | 107 patients with warfarin | Effectiveness and safety: warfarin plasma level, warfarin dose  No significant different were found in various CYP2C19 genotype (C_ss_, CL) | Sandanaraj (2009) |
|  |  | Indonesia | Warfarin 1 mg/day | Cross sectional | 103 patients with warfarin | Effectiveness and safety: PT-INR  No significant PT-INR difference were found in various CYP2C19 genotype | Rusdiana (2013) |
| 4 | Antidiabetic | Myanmar | Gliclazide | Phase I study | 150 healthy subjects | Effectiveness and safety: gliclazide plasma level  Patient with *1/*2 and *2/*2 has significantly higher C_max_, T_max_, AUC, and T_1/2_ compared to *1/*1  Patient *2/*2 has significantly higher C_max_ and AUC | Win (2016) |
| 5 | Antineoplastic | Singapore | Tamoxifen 20 mg for 8 weeks or more | Cross sectional | 165 patients with breast cancer | Effectiveness and safety: pharmacokinetic parameter  No significant difference in tamoxifen, NDM, 4-OHT, and endoxifen level | Lim (2011) |
|  |  |  | Tamoxifen 20 mg for 8 weeks or more | Cross sectional | 201 patients with breast cancer | Effectiveness and safety: pharmacokinetic parameter  CYP2C19*2 variant allele had significantly lower norendoxifen concentration | Lim (2016) |
|  |  | Thailand | Tamoxifen 20 mg for 5 years | Cohort retrospective | 57 patients with breast cancer | Effectiveness: disease-free survival  CYP2C19*2: HR 1.11 (0.55 – 2.23), p: 0.764 | Chamnanphon (2013) |
| 6 | Immunosuppressant | Thailand | Cyclophosphamide | Case control | 36 SLE women with ovarian toxicity  Control: 35 | Safety: ovarian toxicity  CYP2C19*1/*1:  toxicity 17, no toxicity 20  CYP2C19*1/*2:  toxicity 18, no toxicity 15  CYP2C19*2/*2:  toxicity 1, no toxicity 0  CYP2C19*1/*1 genotype and higher dose (>23.75 g) has higher odds; 11.0 (95% CI: 1.2–99.1) | Ngamjanyaporn (2011) |
| 7 | Anticonvulsant | Vietnam | Mephenytoin | Pharmacokinetic analysis | 74 healty Vietnamese subjects | Effectiveness and Safety: S-4’-OH-mephenytoin/S-mephenytoin 4-h plasma concentration ratio after oral intake of 100 mg mephenytoin:  *1/*1: 3.85 (2.87–4.83)  *1/*2: 1.27 (0.95–1.59)  *1/*3: 1.77 (0.71–2.84); p<0.001 | Veiga (2008) |
|  |  | Thailand | Phenobarbital (PB), phenytoin (PHT), and carbamazepine (CBZ) | Case control | 40 children with SCAR (20 in PB, 17 in PHT, 3 in CBZ)  Control: 40 | Safety: SCARs  CYP2C19*1/*1: SCAR 23, no SCAR 31  CYP2C19*1/*2: SCAR 16, no SCAR 7  CYP2C19*2/*2: SCAR 1, no SCAR 2  *2 odds ratio: 2.54 (0.96 to 6.73), p = 0.059 | Manuyakorn  (2013) |
|  |  |  | Phenytoin | Case control | 15 adults with SCAR  Control: 100 PHT-tolerant control | Safety: SCARs  CYP2C19 with *3:  SCAR 5, no SCAR 10  CYP2C19 without *3:  SCAR 10, no SCAR 90  *3 odds ratio: 4.50 (1.28 to 15.81), p = 0.019 | Yampayon (2017) |
| 8 | Antifungal | Thailand | Voriconazole (various dose) | Cross sectional | 285 subjects with fungal infection | Effectiveness and safety: voriconazole plasma level (µg/mL)  EM: 1.470 (0.632 - 2.720)  IM: 1.860 (0.908- 3.030), p = 0.153  PM: 1.900 (1.130 - 3.673), p = 0.039 | Chuwongwattana (2016) |
|  |  |  |  | Cross sectional | 31 subjects <12 years with fungal infection | Effectiveness and safety: voriconazole plasma level (µg/mL)  CYP2C19*1/*1: 1.130 (0.516 - 3.894)  CYP2C19*1/*2: 4.271 (2.066 - 7.716);  p = 0.038  CYP2C19*1/*3: 5.125 (2.959 - 6.040)  CYP2C19*2/*2: 3.793 (2.436 - 4.008)  CYP2C19*2/*3: 2.523 | Chuwongwattana (2020) |
|  |  |  |  |  | 146 subjects >12 years with fungal infection | CYP2C19*1/*1: 2.020 (0.236-0.856)  CYP2C19*1/*2: 2.340 (0.205-0.980)  CYP2C19*1/*3: 2.380 (0.117-1.606)  CYP2C19*2/*2: 2.703 (0.213-1.116)  CYP2C19*2/*3: 2.585 (0.252-1.340)  CYP2C19*1/*17: 2.665 (0.250-1.227) |  |
| 9 | Antimalarial drug | Cambodia | Piperaquine | Pharmacokinetic pharmacogenetic modelling | 60 patients with malaria infection | CYP2C19*3 was not found to significantly affect piperaquine elimination | Hodel (2013) |
|  |  | Thailand | Chloroquine 25 mg/kg over 3 days, followed by 0.3 mg/kg primaquine for 14 days | Cohort prospective (9 months) | 51 patients with *P. vivax* infection | Effectiveness: relapse incidence  NM: relapse 0, no relapse 23  IM: relapse 3, no relapse 19  PM: relapse 1, no relapse 5  *1/*2 + *2/*2: relapse 4, no relapse 18, p = 0.075 | Chamnanphon (2020) |
| 10 | Antiretroviral | Cambodia | Nevirapine | Pharmacokinetic study | 129 HIV patients with nevirapine | Effectiveness and safety: nevirapine clearance  There was no association between CYP2C19 rs4244285 and nevirapine clearance (β = 0.03, P = 0.68) | Bertrand  (2012) |
| 11 | Antibacterial | Thailand | Dapsone | Cross sectional | 16 patients with SCARs  Control: 40 | Safety: SCARs  No significant interaction were found between CYP2C19 variability and incidence of Dapsone induced SCARs. | Satapornpong (2021) |

# Supplementary Table 5. Risk of bias among included study

| **Authors** | **Q1** | **Q2** | **Q3** | **Q4** | **Q5** | **Q6** | **Q7** | **Q8** | **% Yes** | **Risk** |
| --- | --- | --- | --- | --- | --- | --- | --- | --- | --- | --- |
| Kothary (2021) | Y | Y | Y | Y | N | N | Y | Y | 75 | Low |
| Ang (2016) | Y | Y | Y | Y | Y | Y | NA | NA | 75 | Low |
| Areesinpitak (2020) | Y | Y | Y | Y | Y | Y | NA | NA | 75 | Low |
| Aumpan (2020) | Y | Y | N | U | U | U | NA | NA | 25 | High |
| Auttajaroon (2019) | Y | Y | Y | U | U | U | Y | Y | 62.5 | Moderate |
| Chamnanphon (2013) | Y | Y | Y | Y | Y | Y | Y | Y | 100 | Low |
| Chan (2012) | Y | Y | Y | Y | U | N | Y | Y | 75 | Low |
| Chamnanphon (2020) | Y | Y | Y | Y | N | N | Y | Y | 75 | Low |
| Chotivitayatarakorn (2017) | Y | Y | N | U | N | N | Y | Y | 50 | Moderate |
| Chunlertlith (2017) | Y | Y | Y | Y | Y | U | Y | Y | 87.5 | Low |
| Chuwongwattana (2020) | Y | Y | Y | Y | U | U | Y | Y | 75 | Low |
| Goh (2019) | Y | Y | Y | Y | U | N | NA | NA | 50 | Moderate |
| Ikawati (2014) | Y | Y | Y | Y | U | N | NA | NA | 50 | Moderate |
| Hodel (2013) | Y | Y | U | Y | U | U | Y | Y | 62.5 | Moderate |
| Jainan (2014) | Y | Y | N | U | N | N | U | Y | 37.5 | High |
| Rochmawati (2021) | Y | Y | Y | N | N | N | U | N | 37.5 | High |
| Jittikon (2016) | Y | Y | N | Y | U | U | NA | NA | 37.5 | High |
| John (2024) | Y | Y | Y | Y | Y | U | NA | NA | 62.5 | Moderate |
| Lee (2007) | Y | Y | Y | Y | U | U | NA | NA | 50 | Moderate |
| Lim (2010) | Y | Y | Y | Y | N | N | Y | Y | 75 | Low |
| Lim (2016) | Y | Y | Y | N | Y | Y | Y | Y | 87.5 | Low |
| Manuyakorn (2013) | Y | Y | U | N | N | N | Y | Y | 50 | Moderate |
| Mauleekoonphairoj (2020) | Y | Y | Y | Y | U | U | NA | NA | 50 | Moderate |
| Medhasi (2016) | Y | Y | Y | Y | U | N | NA | NA | 50 | Moderate |
| Mejin (2013) | Y | Y | Y | Y | U | N | Y | Y | 75 | Low |
| Miftahussurur (2021) | Y | Y | Y | Y | U | N | Y | Y | 75 | Low |
| Nakhonsri (2024) | Y | Y | Y | Y | Y | Y | NA | NA | 75 | Low |
| Ngamjanyaporn (2011) | Y | Y | Y | N | N | N | Y | Y | 62.5 | Moderate |
| Nun-anan (2015) | Y | Y | Y | Y | N | N | NA | NA | 50 | Moderate |
| Phiphatpatthamaamphan (2016) | Y | Y | N | U | N | N | Y | Y | 50 | Moderate |
| Poonyam (2019) | Y | Y | N | U | N | N | Y | Y | 50 | Moderate |
| Prapitpaiboon (2015) | Y | Y | N | U | N | N | Y | Y | 50 | Moderate |
| Prasertpetmanee (2013) | Y | Y | N | U | U | U | Y | Y | 50 | Moderate |
| Ram (2018) | Y | Y | N | N | N | N | U | Y | 37.5 | High |
| Goh (2017) | Y | Y | Y | Y | N | U | NA | NA | 50 | Moderate |
| Hoang (2022) | Y | Y | Y | Y | N | N | NA | NA | 50 | Moderate |
| Brunham (2014) | Y | Y | Y | Y | Y | Y | NA | NA | 62.5 | Moderate |
| Chuwongwattana (2016) | Y | Y | Y | U | U | N | NA | NA | 37.5 | High |
| Liem (2022) | Y | Y | Y | U | U | N | NA | NA | 37.5 | High |
| Sukmawan (2021) | Y | Y | Y | U | U | N | NA | NA | 37.5 | High |
| Sandanaraj (2009) | Y | Y | Y | Y | Y | Y | NA | NA | 75 | Low |
| Hidayat (2023) | Y | Y | N | N | U | N | U | Y | 37.5 | High |
| Giantini (2023) | Y | Y | Y | U | U | N | NA | NA | 37.5 | High |
| Rusdiana (2013) | Y | Y | Y | Y | Y | N | NA | NA | 62.5 | Moderate |
| Sirivarasai J (2021) | Y | Y | Y | N | U | N | NA | NA | 37.5 | High |
| Bertrand (2012) | Y | Y | Y | Y | Y | N | Y | Y | 87.5 | Low |
| Jirungda (2019) | Y | Y | Y | N | N | N | NA | NA | 37.5 | High |
| Pussadhamma (2018) | Y | Y | Y | Y | U | N | Y | Y | 75 | Low |
| Rahmatini (2018) | Y | Y | Y | Y | U | N | Y | NA | 62.5 | Moderate |
| Sani (2013) | Y | Y | Y | Y | U | N | Y | Y | 75 | Low |
| Soon GH (2021) | Y | Y | Y | Y | Y | Y | Y | Y | 100 | Low |
| Satapornpong (2021) | Y | Y | Y | Y | U | N | Y | Y | 75 | Low |
| Srinarong (2013) | Y | Y | N | N | U | N | NA | NA | 25 | High |
| Suksem (2013) | Y | Y | Y | Y | U | N | NA | NA | 50 | Moderate |
| Sukprasong (2021) | Y | Y | Y | Y | U | N | NA | NA | 50 | Moderate |
| Tan (2017) | Y | Y | N | Y | U | Y | Y | Y | 75 | Low |
| Tan (2019) | Y | Y | N | Y | U | Y | Y | Y | 75 | Low |
| Tassaneeyakul (2002) | Y | Y | Y | Y | U | N | NA | NA | 50 | Moderate |
| Tassaneeyakul (2006) | Y | Y | Y | Y | Y | N | NA | NA | 62.5 | Moderate |
| Thuy (2024) | Y | Y | Y | Y | U | N | Y | Y | 75 | Low |
| Tresukosol (2014) | Y | Y | Y | Y | U | N | Y | Y | 75 | Low |
| Tube (2021) | Y | Y | Y | Y | Y | Y | NA | NA | 75 | Low |
| Veiga (2008) | Y | Y | Y | Y | U | N | Y | Y | 75 | Low |
| Vu (2019) | Y | Y | Y | Y | Y | Y | NA | NA | 75 | Low |
| Wankaew (2022) | Y | Y | Y | Y | U | Y | NA | NA | 62.5 | Moderate |
| Win (2016) | Y | Y | Y | Y | U | N | Y | Y | 75 | Low |
| Yamada (2001) | Y | Y | Y | Y | U | N | NA | NA | 50 | Moderate |
| Yampayon (2017) | Y | Y | Y | Y | U | N | Y | Y | 75 | Low |
| Yang (2004) | Y | Y | Y | Y | U | N | NA | NA | 50 | Moderate |
| Yusaff (2015) | Y | Y | Y | Y | Y | Y | NA | NA | 75 | Low |
| Zumaraga (2022) | Y | Y | Y | Y | U | N | NA | NA | 50 | Moderate |

# Supplementary Table 6. Detailed description on CYP2C19 allele frequency and phenotype in Southeast Asian population

| **No.** | **Country** | **Ethnicity** | **Total Subject** | **CYP2C19 allele frequency** | | | | | | **CYP2C19 phenotype** | | | | | | | | **Reference** |
| --- | --- | --- | --- | --- | --- | --- | --- | --- | --- | --- | --- | --- | --- | --- | --- | --- | --- | --- |
|  |  |  |  | ***2** | | ***3** | | ***17** | | **UM** | | **RM** | | **IM** | | **PM** | |  |
|  |  |  |  | **n** | **f** | **n** | **f** | **n** | **f** | **n** | **%** | **n** | **%** | **n** | **%** | **n** | **%** |  |
| 1 | Indonesia | Java | 151 | 53 | 0.18 | 11 | 0.03 | - | - | - | - | - | - | 46 | 30.5 | 9 | 5.9 | Liem (2022) |
|  |  |  | 28 | 16 | 0.29 | 6 | 0.10 | - | - | - | - | - | - | 8 | 28.6 | 7 | 25 | Miftahussurur (2021) |
|  |  | Buginese | 96 | - | - | - | - | 9 | 0.04 | 0 | 0 | 9 | 9.4 | - | - | - | - | Ikawati (2014) |
|  |  |  | 37 | 25 | 0.34 | 2 | 0.03 | - | - | - | - | - | - | 19 | 51.4 | 4 | 10.8 | Miftahussurur (2021) |
|  |  | Various | 30 | 17 | 0.28 | 4 | 0.07 | - | - | - | - | - | - | 12 | 40.0 | 4 | 13.3 | Rahmatini (2018) |
|  |  |  | 166 | 76 | 0.22 | 60 | 0.18 | - | - | - | - | - | - | 44 | 26.5 | 46 | 27.7 | Sukmawan (2021) |
|  |  |  | 206 | 86 | 0.41 | 19 | 0.09 | - | - | - | - | - | - | 73 | 35.4 | 7 | 3.3 | Rusdia- (2013) |
|  |  |  | 53 | - | - | - | - | - | - | - | - | - | - | - | - | - | - | Rochmawati (2021) |
|  |  |  | 37 | 22 | 0.29 | 3 | 0.04 | 4 | 0.05 | 0 | 0 | 3 | 8.1 | 23 | 62.2 | 1 | 2.7 | Kothary (2021) |
|  |  |  | 112 | - | - | - | - | - | - | - | - | - | - | - | - | - | - | Hidayat (2023) |
|  |  | Papua | 99 | - | - | - | - | 0 | 0 | 0 | 0 | 0 | 0 | - | - | - | - | Tuba (2021) |
|  |  |  | 14 | 15 | 0.54 | 5 | 0.18 | - | - | - | - | - | - | 4 | 28.6 | 8 | 57.1 | Miftahussurur (2021) |
|  |  | Batak | 27 | 16 | 0.29 | 5 | 0.09 | - | - | - | - | - | - | 13 | 48.1 | 4 | 14.8 | Miftahussurur (2021) |
|  |  | Balinese | 25 | 16 | 0.32 | 1 | 0.02 | - | - | - | - | - | - | 8 | 32 | 4 | 16 | Miftahussurur (2021) |
|  |  | Dayak | 10 | 7 | 0.35 | 1 | 0.05 | - | - | - | - | - | - | 4 | 40 | 2 | 20 | Miftahussurur (2021) |
|  |  | Chinese | 17 | 13 | 0.38 | 3 | 0.09 | - | - | - | - | - | - | 8 | 47.1 | 4 | 23.5 | Miftahussurur (2021) |
|  |  | Timorese | 8 | 5 | 0.31 | 0 | 0 | - | - | - | - | - | - | 5 | 62.5 | 0 | 0 | Miftahussurur (2021) |
| 2 | Cambodia | Various | 129 | 71 | 0.27 | - | - | - | - | - | - | - | - | - | - | - | - | Bertrand (2012) |
|  |  |  | 18 | - | - | - | - | - | - | - | - | - | - | 7 | 38.9 | 1 | 5.5 | Aumpan (2020) |
|  |  |  | 123 | - | - | 7 | 0.03 | - | - | - | - | - | - | - | - | - | - | Hodel (2013) |
| 3 | Thailand | Various | 115 | 60 | 0.26 | 12 | 0.05 | 0 | 0 | 0 | 0 | 0 | 0 | 42 | 36.5 | 14 | 12.2 | Chuwongwattana (2016) |
|  |  |  | 102 | 96 | 0.47 | - | - | - | - | - | - | - | - | - | - | - | - | Jirungda (2019) |
|  |  |  | 67 | - | - | - | - | - | - | - | - | - | - | 32 | 47.8 | 9 | 13.4 | Pussadhamma (2018) |
|  |  |  | 445 | 261 | 0.29 | - | - | - | - | - | - | - | - | - | - | - | - | Sirivarasai (2021) |
|  |  |  | 72 | 43 | 0.30 | 9 | 0.06 | 2 | 0.017 | - | - | 2 | 2.7 | 36 | 50 | 8 | 11.1 | Satapornpong (2021) |
|  |  |  | 98 | - | - | - | - | - | - | - | - | - | - | 48 | 49 | 10 | 10.2 | Srinarong (2013) |
|  |  |  | 1051 | 568 | 0.27 | 126 | 0.06 | 90 | 0.04 | - | - | 45 | 4.30 | 441 | 41.9 | 137 | 13.0 | Sukasem (2013) |
|  |  |  | 1250 | 617 | 0.26 | 59 | 0.03 | 43 | 0.018 | - | - | 26 | 2.16 | 518 | 42.9 | 80 | 6.6 | Sukprasong (2021) |
|  |  |  | 107 | 57 | 0.27 | 5 | 0.02 | - | - | - | - | - | - | 50 | 46.7 | 6 | 5.6 | Tassaneeyakul (2002) |
|  |  |  | 211 | 114 | 0.27 | 13 | 0.03 | - | - | - | - | - | - | 82 | 38.9 | 23 | 10.9 | Tresukosol (2014) |
|  |  |  | 171 | - | - | - | - | - | - | 1 | 0.5 | 2 | 1.16 | 77 | 45 | 24 | 14 | Wankaew (2022) |
|  |  |  | 121 | 85 | 0.46 | 12 | 0.05 | - | - | - | - | - | - | 81 | 47.1 | 19 | 15.7 | Yamada ( 2001) |
|  |  |  | 136 | - | - | 10 | 0.04 | - | - | - | - | - | - | - | - | - | - | Yampayon (2017) |
|  |  |  | 93 | - | - | - | - | - | - | - | - | - | - | 38 | 40.9 | 3 | 3.2 | Auttajaroon (2019) |
|  |  |  | 57 | 27 | 0.24 | 6 | 0.05 | - | - | - | - | - | - | 27 | 47.4 | 3 | 5.3 | Chamnanphon (2013) |
|  |  |  | 51 | - | - | - | - | - | - | 0 | 0 | 0 | 0 | 21 | 41.1 | 6 | 11.8 | Chamnanphon (2020) |
|  |  |  | 103 | - | - | - | - | - | - | - | - | - | - | 48 | 46.6 | 11 | 10.7 | Chotivitayatarakorn (2017) |
|  |  |  | 170 | - | - | - | - | - | - | - | - | - | - | 78 | 45.9 | 16 | 9.4 | Chunlertlith (2017) |
|  |  |  | 177 | 103 | 0.29 | 21 | 0.06 | 3 | 0.01 | 0 | 0 | 2 | 1.1 | 83 | 46.9 | 20 | 11.3 | Chuwongwattana (2020) |
|  |  |  | 202 | - | - | - | - | - | - | - | - | - | - | 99 | 49 | 21 | 10.4 | Jainan (2014) |
|  |  |  | 190 | 99 | 0.26 | 12 | 0.03 | 19 | 0.05 | - | - | - | - | - | - | - | - | Jittikon (2016) |
|  |  |  | 942 | 503 | 0.27 | 78 | 0.04 | - | - | - | - | - | - | 400 | 42.5 | 93 | 9.9 | John (2024) |
|  |  |  | 64 | - | - | - | - | - | - | - | - | - | - | 19 | 29.7 | 9 | 14.1 | Prasertpetmanee (2013) |
|  |  |  | 100 | - | - | - | - | - | - | - | - | - | - | 50 | 50 | 13 | 13 | Poonyam (2019) |
|  |  |  | 98 | - | - | - | - | - | - | - | - | - | - | 48 | 49 | 6 | 6.1 | Phiphatpatthamaamphan (2016) |
|  |  |  | 100 | - | - | - | - | - | - | - | - | - | - | 50 | 50 | 10 | 10 | Nun-anan (2015) |
|  |  |  | 71 | 35 | 0.25 | - | - | - | - | - | - | - | - | - | - | - | - | Ngamjanyaporn (2011) |
|  |  |  | 119 | 79 | 0.34 | 12 | 0.05 | - | - | - | - | - | - | 57 | 47.6 | 28 | 23.5 | Medhasi (2016) |
|  |  |  | 291 | 163 | 0.28 | 15 | 0.03 | 7 | 0.01 | 0 | 0 | 7 | 2.4 | 114 | 39.2 | 32 | 11 | Mauleekoonphairoj (2020) |
|  |  |  | 80 | 29 | 0.18 | - | - | - | - | - | - | - | - | - | - | - | - | Manuyakorn (2013) |
|  |  |  | 98 | - | - | - | - | - | - | - | - | - | - | 34 | 34.7 | 11 | 11.2 | Prapitpaiboon (2015) |
|  |  | Northeastern Thai | 249 | 119 | 0.24 | 15 | 0.03 | 10 | 0.02 | - | - | 6 | 2.4 | 96 | 38.6 | 19 | 7.6 | Areesinpitak (2020) |
|  |  | Thais | 774 | 449 | 0.29 | 47 | 0.03 | - | - | - | - | - | - | 359 | 46.4 | 71 | 9.1 | Tassaneeyakul (2006) |
|  |  |  | 211 | 107 | 0.25 | 19 | 0.05 | - | - | - | - | - | - | 90 | 42.7 | 18 | 8.5 | Nakhonsri (2024) |
|  |  | Burmeses | 127 | 76 | 0.30 | 10 | 0.04 | - | - | - | - | - | - | 57 | 44.9 | 14 | 8.7 | Tassaneeyakul (2006) |
|  |  | Karens | 131 | 74 | 0.28 | 3 | 0.01 | - | - | - | - | - | - | 53 | 40.5 | 11 | 8.4 | Tassaneeyakul (2006) |
| 4 | Singapore | Chinese | 88 | - | - | 11 | 0.06 | - | - | - | - | - | - | - | - | - | - | Brunham (2014) |
|  |  |  | 107 | 53 | 0.25 | 14 | 0.07 | - | - | - | - | - | - | 47 | 43.9 | 10 | 9.3 | Sandanaraj (2009) |
|  |  |  | 367 | 233 | 0.32 | 46 | 0.06 | - | - | - | - | - | - | 84 | 11.4 | 53 | 14.4 | Soon (2021) |
|  |  |  | 100 | 56 | 0.28 | 13 | 0.07 | 2 | 0.01 | 0 | 0 | 2 | 2 | 49 | 49 | 10 | 10 | Chan (2012) |
|  |  |  | 201 | 150 | 0.37 | 15 | 0.04 | 4 | 0.001 | 0 | 0 | 2 | 1 | 95 | 47.3 | 34 | 17 | Goh (2017) |
|  |  |  | 73 | 40 | 0.27 | 8 | 0.06 | 1 | 0.007 | 0 | 0 | 1 | 1.4 | 34 | 46.6 | 7 | 9.6 | Kothary (2021) |
|  |  |  | 76 | 48 | 0.32 | 11 | 0.07 | 1 | 0.007 | 0 | 0 | 1 | 1.3 | 39 | 51.3 | 10 | 13.2 | Lim (2011) |
|  |  |  | 80 | 48 | 0.3 | 8 | 0.05 | 2 | 0.013 | 0 | 0 | 1 | 1.7 | 37 | 45.8 | 10 | 12.3 | Lim (2016) |
|  |  | Malay | 87 | - | - | 8 | 0.05 | - | - | - | - | - | - | - | - | - | - | Brunham (2014) |
|  |  |  | 43 | 25 | 0.29 | 3 | 0.03 | - | - | - | - | - | - | 19 | 43.8 | 5 | 10.5 | Soon (2021) |
|  |  |  | 100 | 62 | 0.31 | 10 | 0.05 | 5 | 0.03 | 0 | 0 | 3 | 3 | 48 | 48 | 12 | 12 | Chan (2012) |
|  |  |  | 126 | 69 | 0.27 | 7 | 0.03 | 6 | 0.02 | 0 | 0 | 5 | 4 | 55 | 43.7 | 10 | 8 | Goh (2017) |
|  |  |  | 38 | 22 | 0.29 | 5 | 0.07 | 3 | 0.04 | 1 | 2.63 | 1 | 2.6 | 17 | 44.7 | 5 | 13.2 | Kothary (2021) |
|  |  |  | 76 | 35 | 0.23 | 11 | 0.07 | 7 | 0.05 | 0 | 0 | 7 | 9.2 | 40 | 52.6 | 3 | 3.95 | Lim (2011) |
|  |  |  | 80 | 35 | 0.22 | 10 | 0.06 | 16 | 0.1 | 1 | 1 | 10 | 12.4 | 32 | 40.3 | 6 | 7.8 | Lim (2016) |
|  |  | Indian | 78 | - | - | 1 | 0.006 | - | - | - | - | - | - | - | - | - | - | Brunham (2014) |
|  |  |  | 45 | 42 | 0.46 | 0 | 0 | - | - | - | - | - | - | 23 | 49.7 | 10 | 21.2 | Soon (2021) |
|  |  |  | 100 | 65 | 0.33 | 2 | 0.01 | 33 | 0.17 | 5 | 5 | 13 | 13 | 37 | 37 | 15 | 15 | Chan (2012) |
|  |  |  | 179 | 136 | 0.38 | 4 | 0.011 | 54 | 0.15 | 1 | 0.6 | 27 | 15.1 | 81 | 45.3 | 29 | 16.2 | Goh (2017) |
|  |  |  | 24 | 11 | 0.23 | 0 | 0 | 11 | 0.23 | 1 | 4.17 | 5 | 20.8 | 9 | 37.5 | 1 | 4.17 | Kothary (2021) |
|  |  |  | 76 | 58 | 0.38 | 1 | 0.007 | 29 | 0.19 | 3 | 3.95 | 23 | 30.3 | 39 | 51.3 | 10 | 13.2 | Lim (2011) |
|  |  |  | 80 | 58 | 0.36 | 1 | 0.006 | 61 | 0.38 | 12 | 14.4 | 15 | 19.3 | 37 | 46.5 | 11 | 13.4 | Lim (2016) |
|  |  | Various | 165 | 112 | 0.34 | 17 | 0.05 | 11 | 0.03 | 0 | 0 | 11 | 6.7 | 81 | 49.1 | 24 | 14.5 | Lim (2010) |
|  |  | Caucasian | 29 | 8 | 0.14 | 1 | 0.017 | 8 | 0.14 | 1 | 3.45 | 5 | 17.2 | 9 | 31.0 | 0 | 0 | Kothary (2021) |
| 5 | Malaysia | Various | 90 | 54 | 0.30 | 3 | 0.017 | - | - | - | - | - | - | 40 | 44.4 | 16 | 17.8 | Sani (2013) |
|  |  |  | 193 | - | - | - | - | - | - | - | - | - | - | - | - | - | - | Ram (2018) |
|  |  |  | 30 | 20 | 0.33 | - | - | - | - | - | - | - | - | 14 | 46.7 | 3 | 10 | Tan (2017) |
|  |  |  | 8 | 1 | 0.06 | 1 | 0.06 | 4 | 0.25 | 1 | 12.5 | 2 | 25 | 2 | 25 | 0 | 0 | Mejin (2013) |
|  |  | Malay | 34 | 17 | 0.25 | 2 | 0.03 | - | - | - | - | - | - | 14 | 40.3 | 3 | 7.9 | Sani (2013) |
|  |  |  | 60 | 26 | 0.22 | - | - | - | - | - | - | - | - | 20 | 33.3 | 3 | 5 | Tan (2017) |
|  |  |  | 54 | 25 | 0.23 | 6 | 0.05 | - | - | - | - | - | - | 24 | 44.4 | 3 | 5 | Yang (2003) |
|  |  |  | 209 | 84 | 0.20 | 10 | 0.02 | - | - | - | - | - | - | 70 | 33.5 | 12 | 5.7 | Yusoff (2015) |
|  |  |  | 29 | 16 | 0.28 | 6 | 0.10 | 1 | 0.017 | 0 | 0 | 1 | 3.4 | 14 | 48.3 | 4 | 13.8 | Mejin (2013) |
|  |  | Chinese | 49 | 33 | 0.34 | 1 | 0.01 | - | - | - | - | - | - | 22 | 45.5 | 6 | 12.3 | Sani (2013) |
|  |  |  | 68 | 42 | 0.31 | 14 | 0.10 | - | - | - | - | - | - | 30 | 44.1 | 12 | 17.7 | Yang (2003) |
|  |  |  | 122 | 68 | 0.28 | - | - | - | - | - | - | - | - | 44 | 36.1 | 12 | 9.8 | Tan (2017) |
|  |  |  | 201 | 107 | 0.27 | 6 | 0.15 | - | - | - | - | - | - | 65 | 32.3 | 32 | 15.9 | Yusoff (2015) |
|  |  |  | 57 | 43 | 0.38 | 3 | 0.03 | 3 | 0.03 | 0 | 0 | 2 | 3.5 | 28 | 49.1 | 9 | 15.8 | Mejin (2013) |
|  |  | Indian | 7 | 4 | 0.29 | 0 | 0 | - | - | - | - | - | - | 3 | 41.2 | 1 | 8.4 | Sani (2013) |
|  |  |  | 20 | 16 | 0.38 | 0 | 0 | - | - | - | - | - | - | 11 | 55 | 2 | 10 | Yang (2003) |
|  |  |  | 200 | 123 | 0.31 | 8 | 0.02 | - | - | - | - | - | - | 89 | 44.5 | 21 | 10.5 | Yusoff (2015) |
|  |  | Iban | 43 | 21 | 0.24 | - | - | - | - | - | - | - | - | 17 | 39.5 | 2 | 4.6 | Tan (2017) |
|  |  |  | 24 | 8 | 0.17 | 5 | 0.10 | 1 | 0.02 | 0 | 0 | 1 | 4.2 | 9 | 37.5 | 2 | 8.3 | Mejin (2013) |
|  |  | Orang Asli | 176 | 56 | 0.16 | 0 | 0 | - | - | - | - | - | - | 40 | 22.7 | 8 | 4.5 | Yusoff (2015) |
|  |  | Negritos – Bateq | 9 | 0 | 0 | 0 | 0 | 2 | 0.11 | - | - | 2 | 22.2 | 0 | 0 | 0 | 0 | Ang (2016) |
|  |  | Negritos – Kensiu | 10 | 1 | 0.05 | 1 | 0.05 | 1 | 0.05 | - | - | 1 | 10.0 | 2 | 20.0 | 0 | 0 | Ang (2016) |
|  |  | Negritos – Lanoh | 10 | 3 | 0.15 | 0 | 0 | 3 | 0.15 | - | - | 3 | 30.0 | 3 | 30.0 | 0 | 0 | Ang (2016) |
|  |  | Senoi – Che Wong | 13 | 1 | 0.04 | 7 | 0.27 | 0 | 0 | - | - | 0 | 0 | 2 | 15.4 | 3 | 23.1 | Ang (2016) |
|  |  | Senoi – Semai | 14 | 2 | 0.07 | 0 | 0 | 0 | 0 | - | - | 0 | 0 | 2 | 14.3 | 0 | 0 | Ang (2016) |
|  |  | Proto-Malay – Kanaq | 6 | 0 | 0 | 0 | 0 | 0 | 0 | - | - | 0 | 0 | 0 | 0 | 0 | 0 | Ang (2016) |
| 6 | Vietnam | Kinh | 206 | 108 | 0.26 | 7 | 0.02 | 17 | 0.04 | - | - | - | - | 81 | 39.3 | 17 | 8.3 | Hoang (2022) |
|  |  |  | 100 | 41 | 0.21 | 5 | 0.03 | 2 | 0.01 | 0 | 0 | 2 | 1.5 | 4 | 3.8 | 6 | 5.3 | Vu (2019) |
|  |  |  | 165 | 87 | 0.26 | 16 | 0.05 | - | - | - | - | - | - | 79 | 47.9 | 12 | 7.3 | Lee (2007) |
|  |  | Tay | 103 | 79 | 0.38 | 5 | 0.02 | 3 | 0.01 | 0 | 0 | 2 | 1.6 | 50 | 48.4 | 17 | 16.6 | Vu (2019) |
|  |  | Muong | 99 | 67 | 0.34 | 11 | 0.06 | 2 | 0.01 | 0 | 0 | 2 | 1.2 | 48 | 47.8 | 16 | 15.5 | Vu (2019) |
|  |  | H’Mong | 40 | 43 | 0.54 | 0 | 0 | 0 | 0 | 0 | 0 | 0 | 0 | 20 | 49.7 | 12 | 28.8 | Vu (2019) |
|  |  | Nung | 33 | 29 | 0.44 | 3 | 0.05 | 0 | 0 | 0 | 0 | 0 | 0 | 25 | 73.4 | 8 | 23.5 | Vu (2019) |
|  |  | Various | 207 | 122 | 0.29 | 25 | 0.06 | - | - | - | - | - | - | 96 | 45.5 | 28 | 15.9 | Thuy (2024) |
|  |  |  | 74 | 45 | 0.31 | 9 | 0.06 | - | - | - | - | - | - | 35 | 46.6 | 20 | 27.12 | Veiga (2008) |
|  |  |  | 90 | 50 | 0.24 | 25 | 0.14 | - | - | - | - | - | - | 36 | 40 | 18 | 20 | Yamada (2001) |
| 7 | Myanmar | Various | 150 | 80 | 0.27 | - | - | - | - | - | - | - | - | 60 | 40 | 10 | 6.67 | Win (2016) |
| 8 | Philippines | Filipino | 147 | 82 | 0.28 | - | - | - | - | - | - | - | - | - | - | - | - | Zumaraga (2013) |

# Supplementary Table 7. CYP2C19 polymorphism and clinical outcomes based on endoscopy

| CYP2C19 | Gastritis | | Peptic Ulcer | |
| --- | --- | --- | --- | --- |
|  | Jainan 2014 | Miftahussurur 2021 | Jainan 2014 | Miftahussurur 2021 |
| UM, RM, and NM | 41 (36.0) | 55 (38.5) | 41 (47.7) | 1 (16.7) |
| IM | 60 (52.6) | 57 (39.9) | 37 (43.0) | 4 (66.6) |
| PM | 13 (11.4) | 31 (21.7) | 8 (9.3) | 1 (16.7) |
| Total | 114 (100) | 143 (100) | 86 (100%) | 6 (100) |

Data presented in frequency (percent)

a.


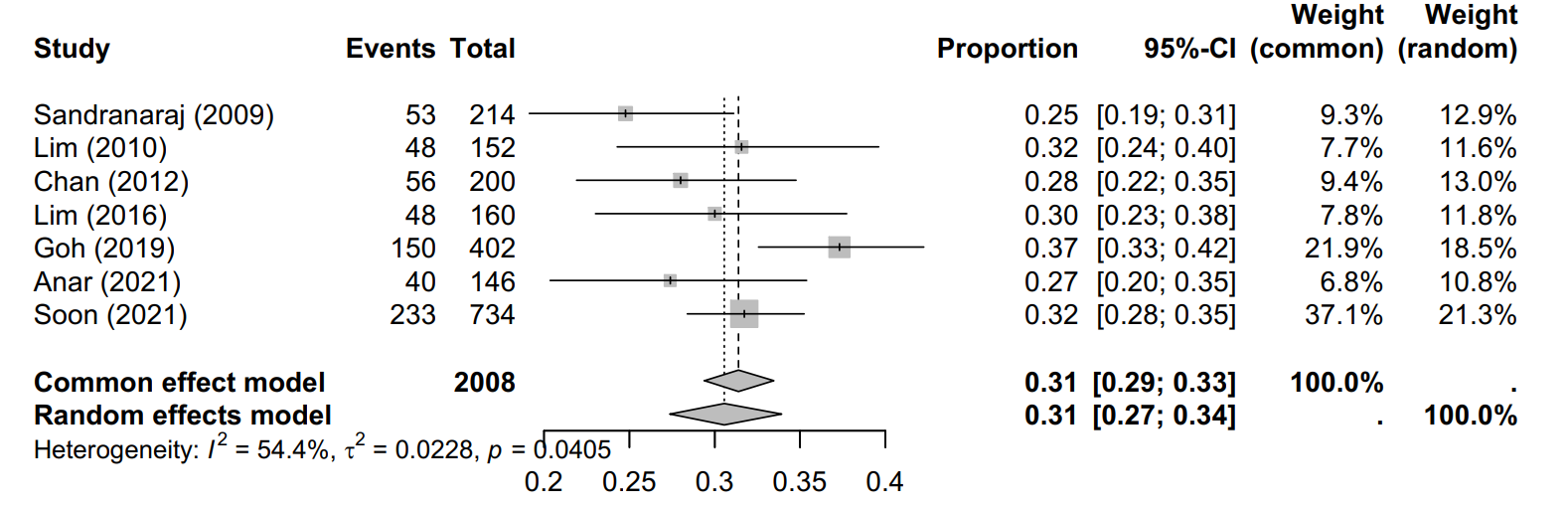


b.


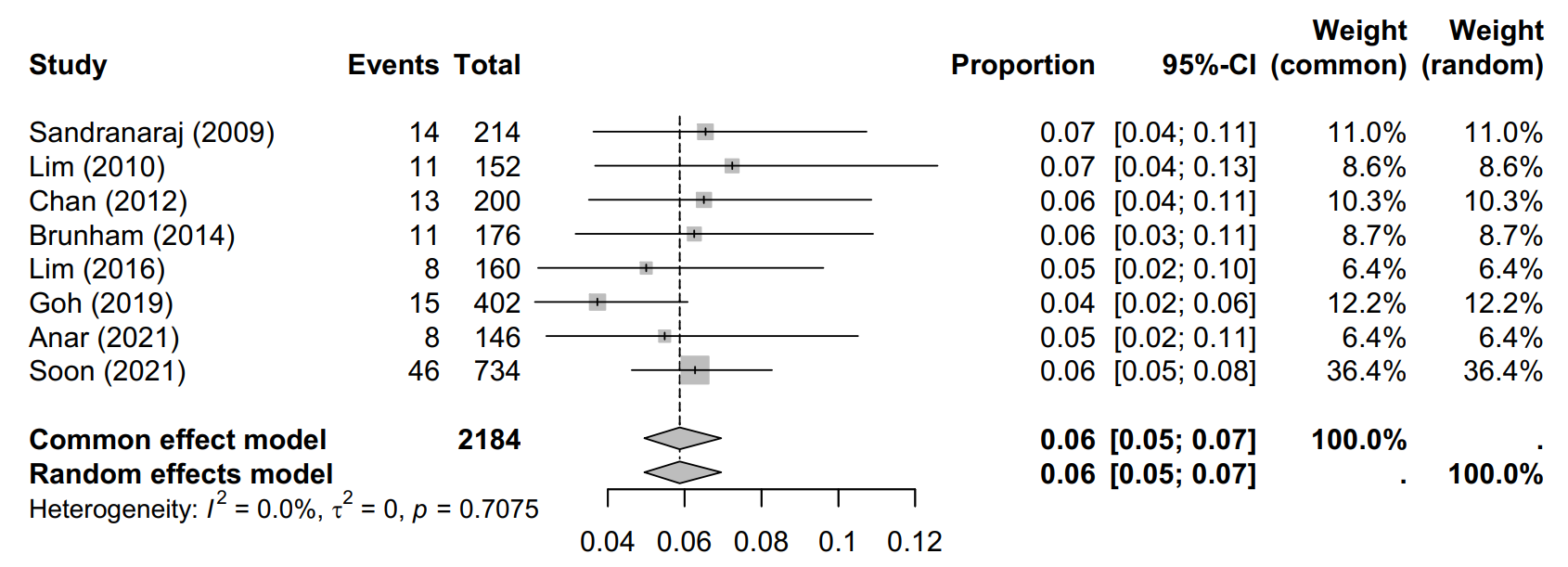


c.


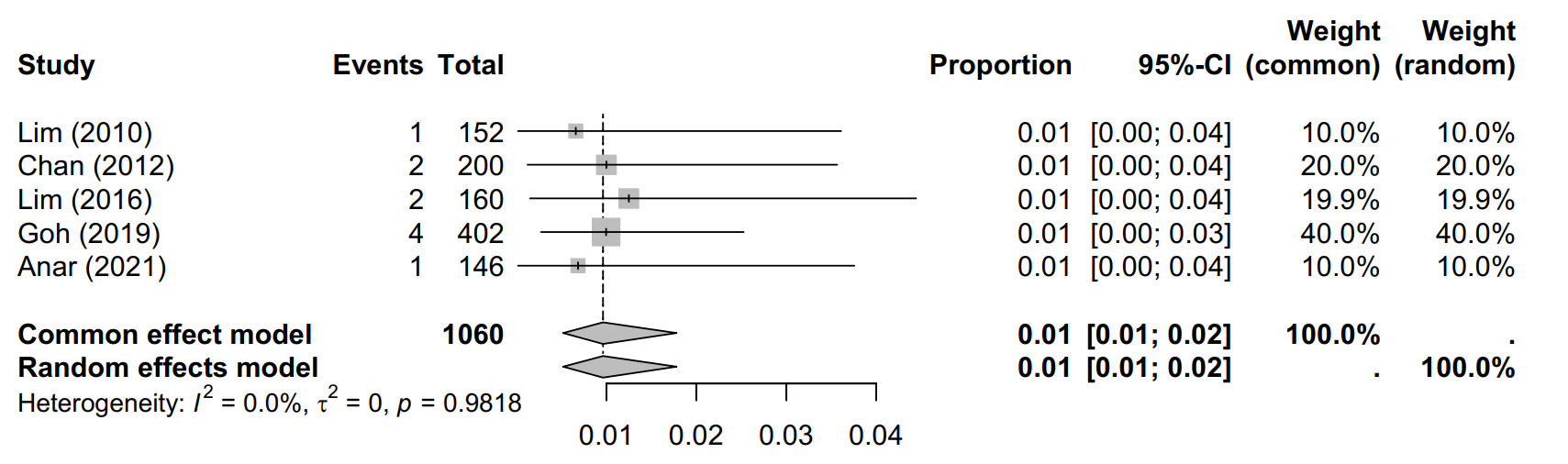


# Supplementary Figure 1. Meta-analysis of allele frequencies in Chinese Singaporeans: (a) for *2 alleles; (b) for *3 alleles; (c) for *17 alleles.

a.


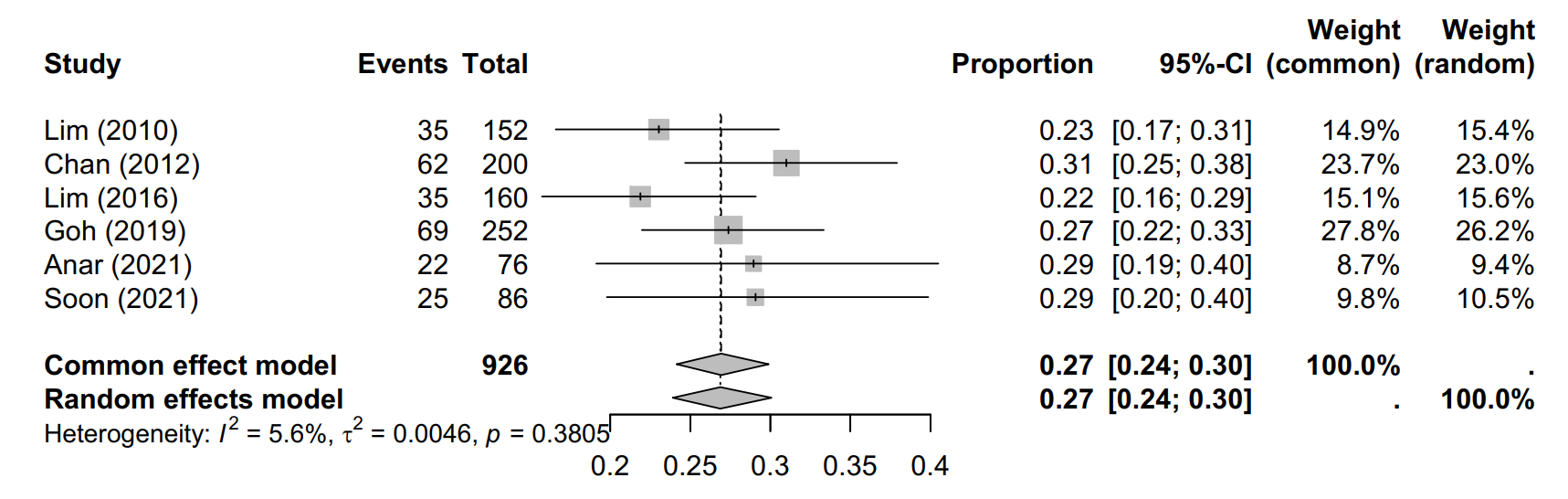


b.


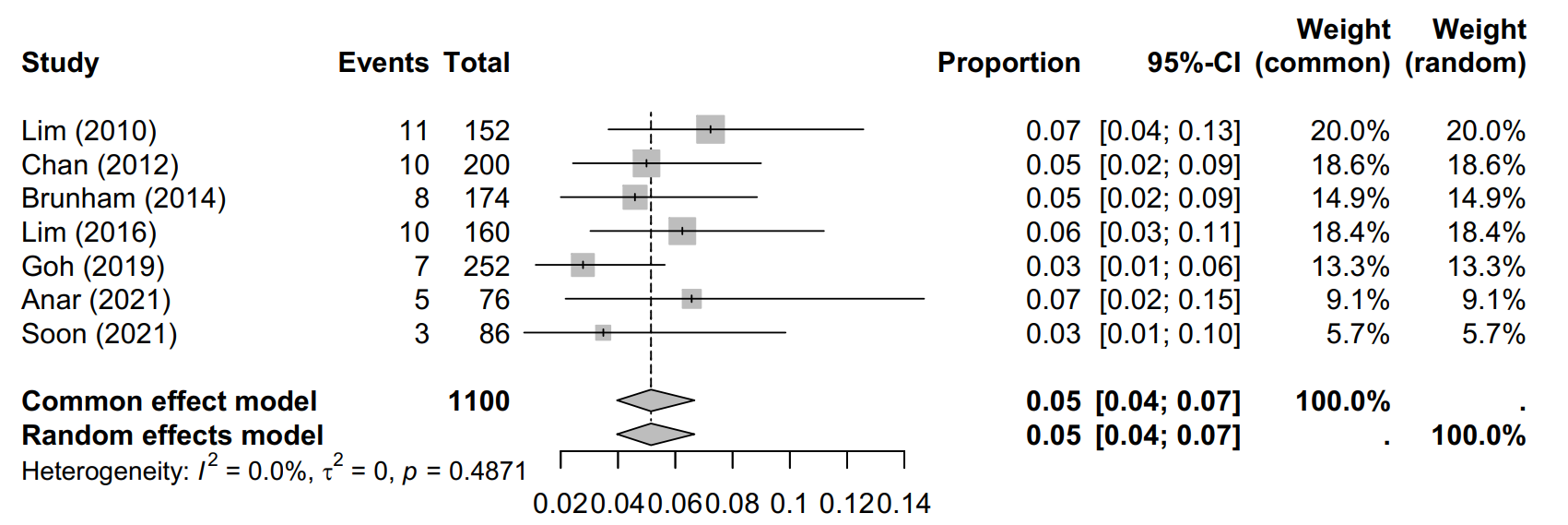


c.


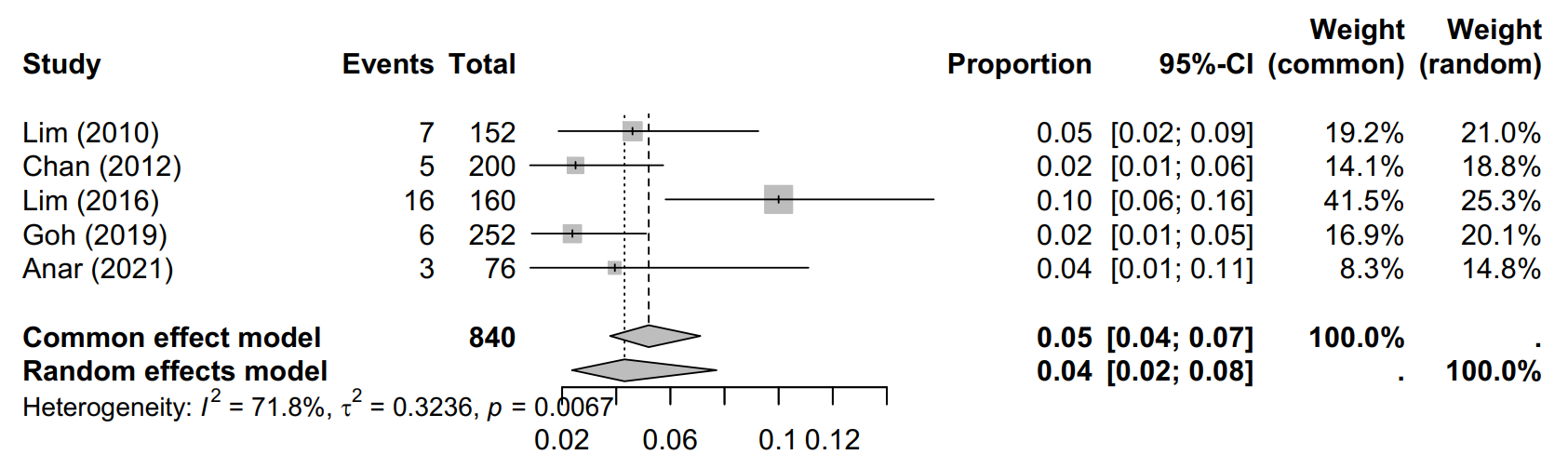


# Supplementary Figure 2. Meta-analysis of allele frequencies in Malay Singaporeans: (a) for *2 alleles; (b) for *3 alleles; (c) for *17 alleles.

a.


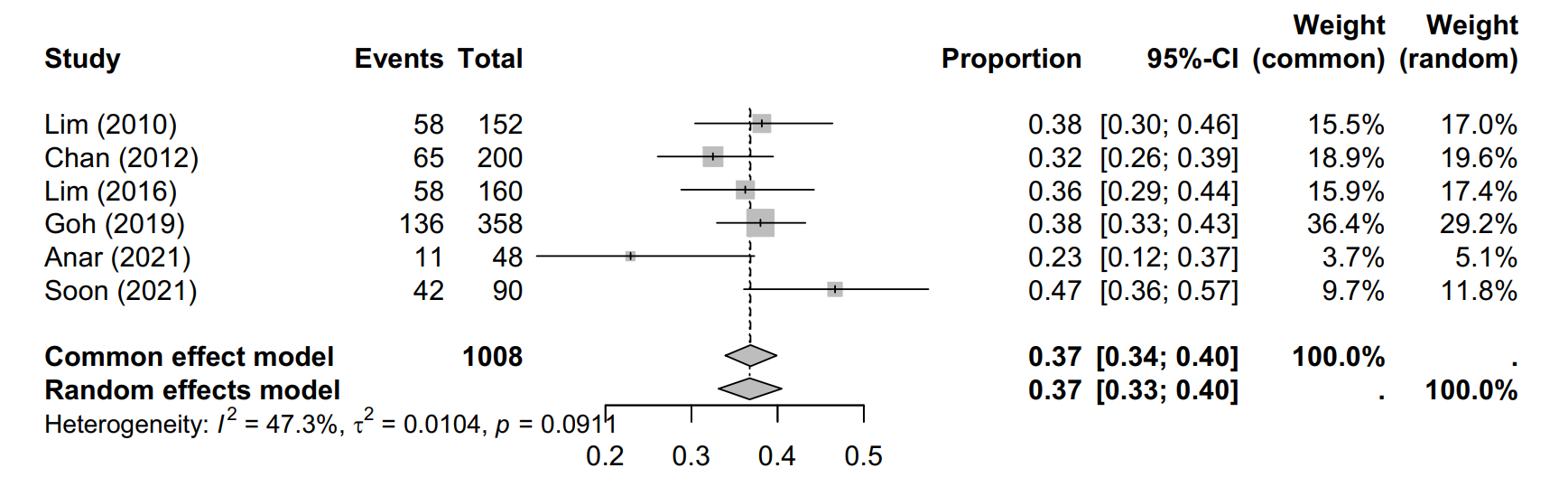
 b.


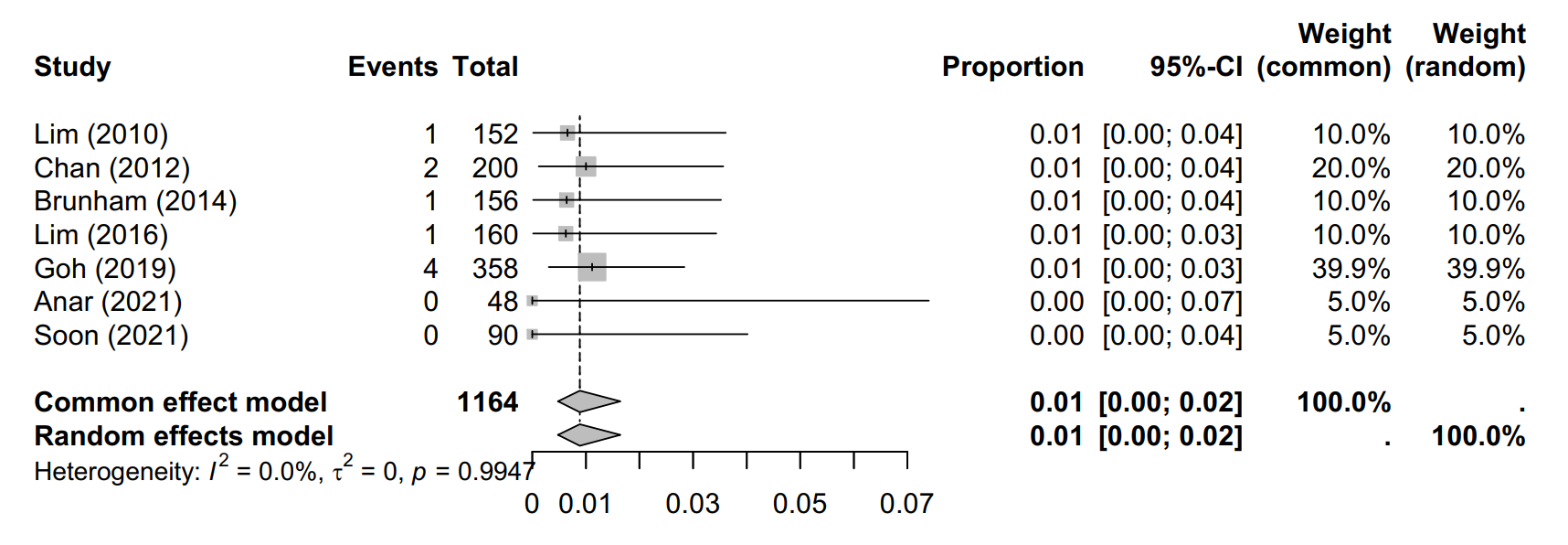


c.


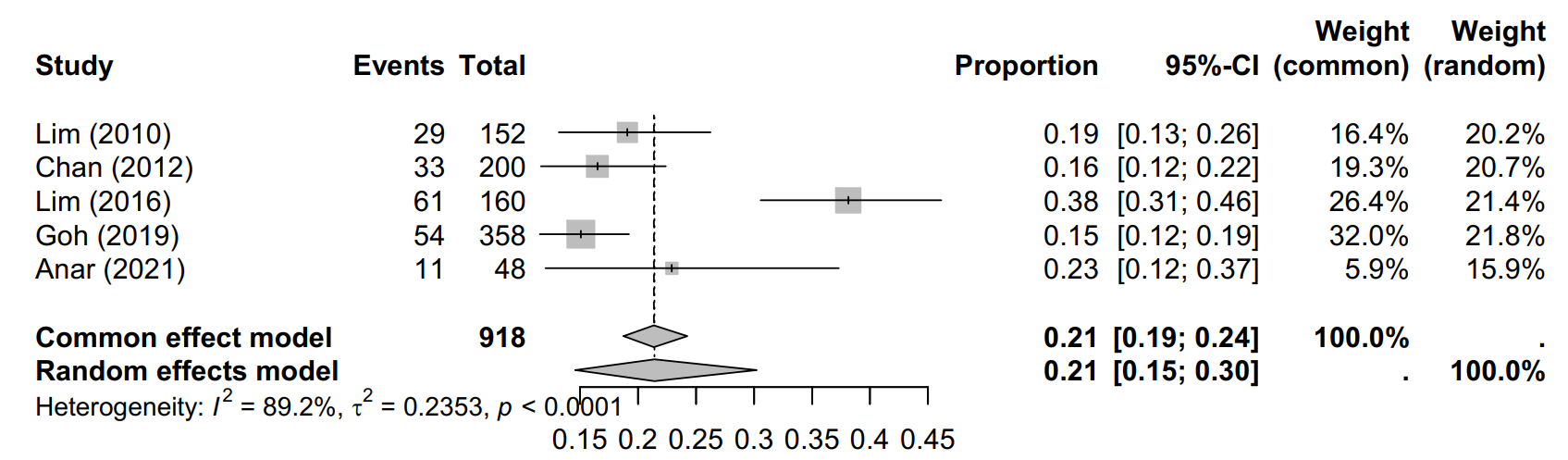


# Supplementary Figure 3. Meta-analysis of allele frequencies in Indian Singaporeans: (a) for *2 alleles; (b) for *3 alleles; (c) for *17 alleles.

a.


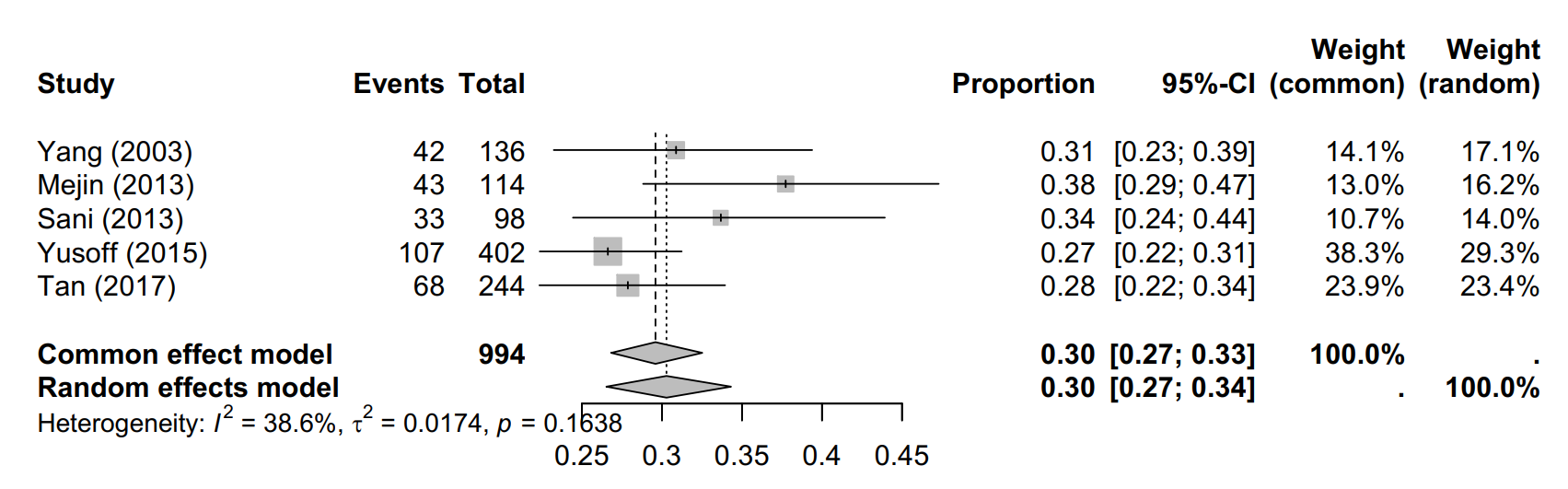


b.
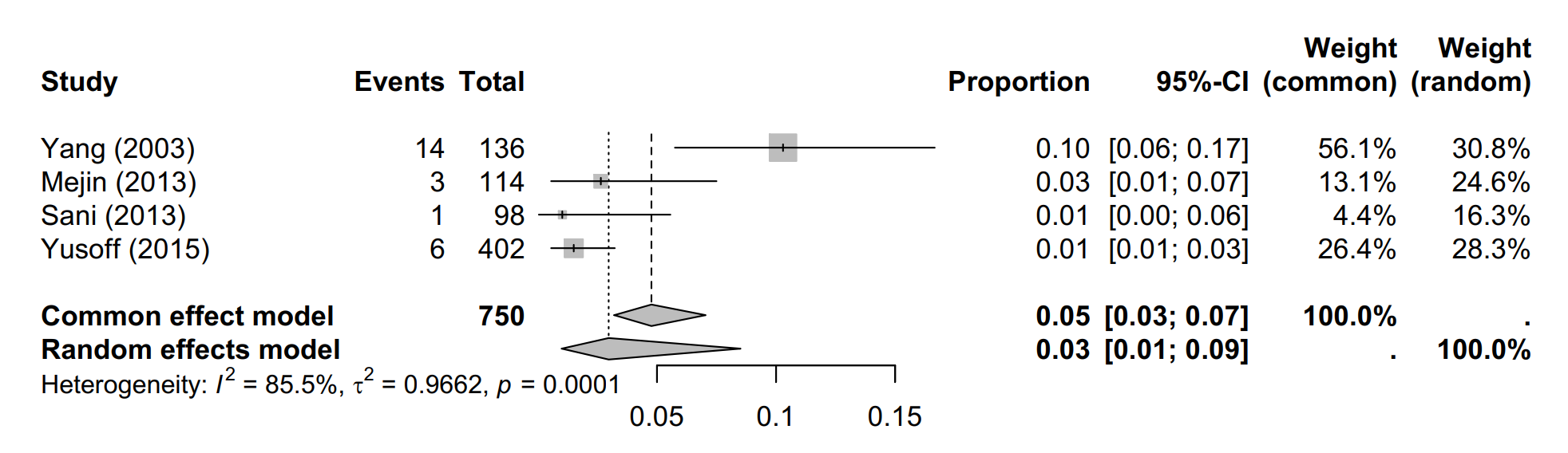


# Supplementary Figure 4. Meta-analysis of allele frequencies in Chinese Malaysians: (a) for *2 alleles; (b) for *3 alleles.

a.


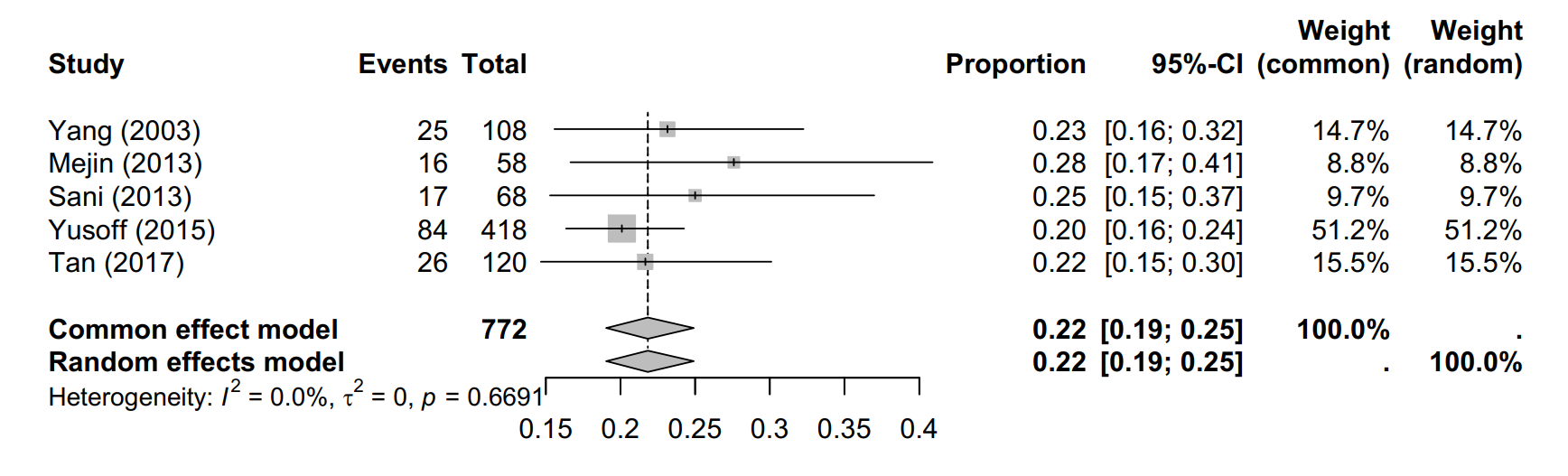


b.


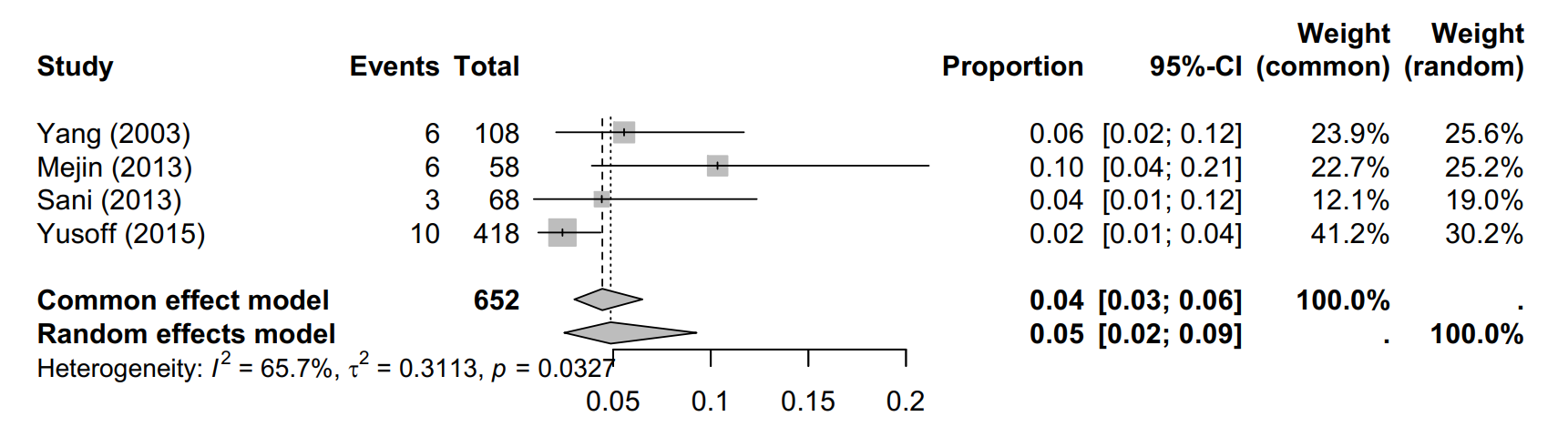


# Supplementary Figure 5. Meta-analysis of allele frequencies in Malay Malaysians: (a) for *2 alleles; (b) for *3 alleles.

a.


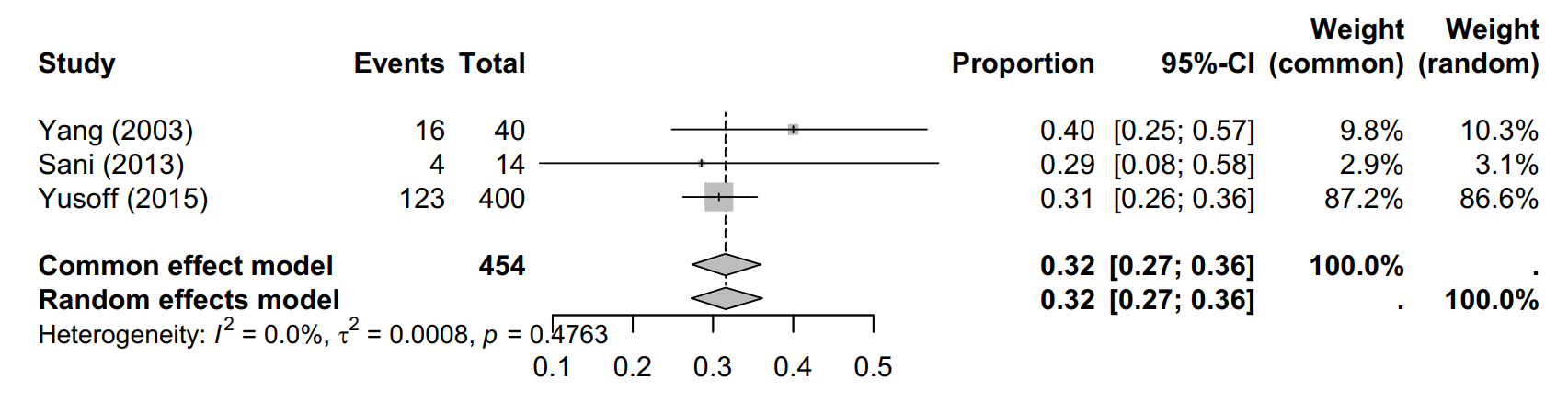


b.


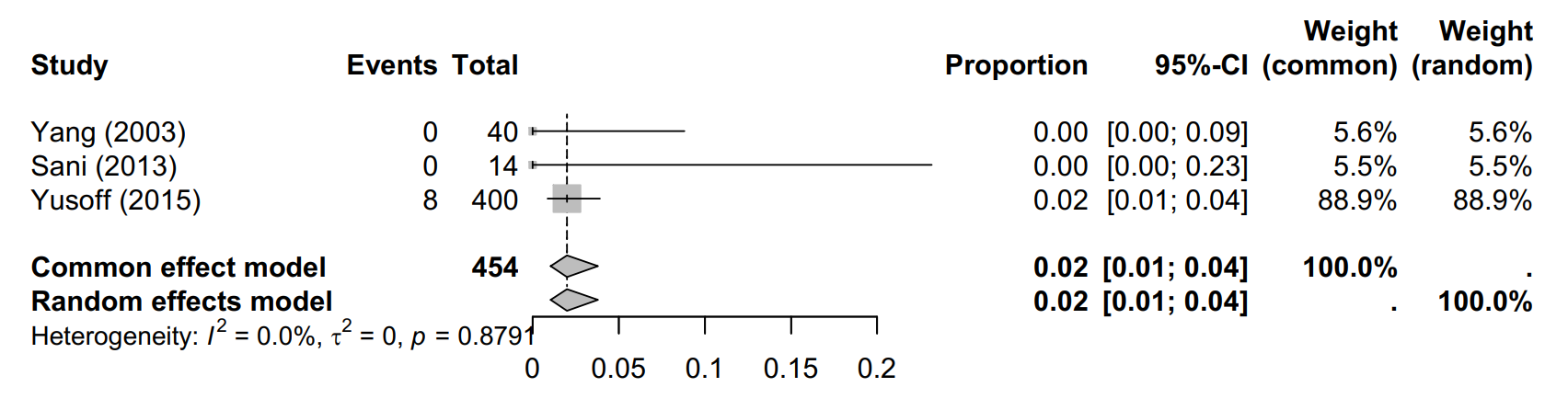


# Supplementary Figure 6. Meta-analysis of allele frequencies in Indian Malaysians: (a) for *2 alleles; (b) for *3 alleles.


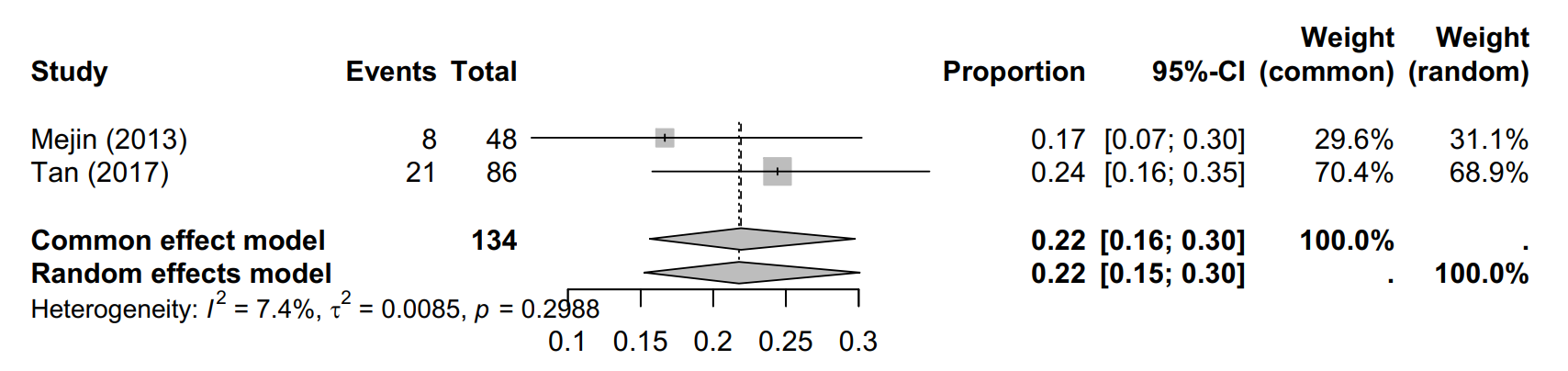


# Supplementary Figure 7. Meta-analysis of allele frequencies in Iban Malaysians for *2 alleles

a.


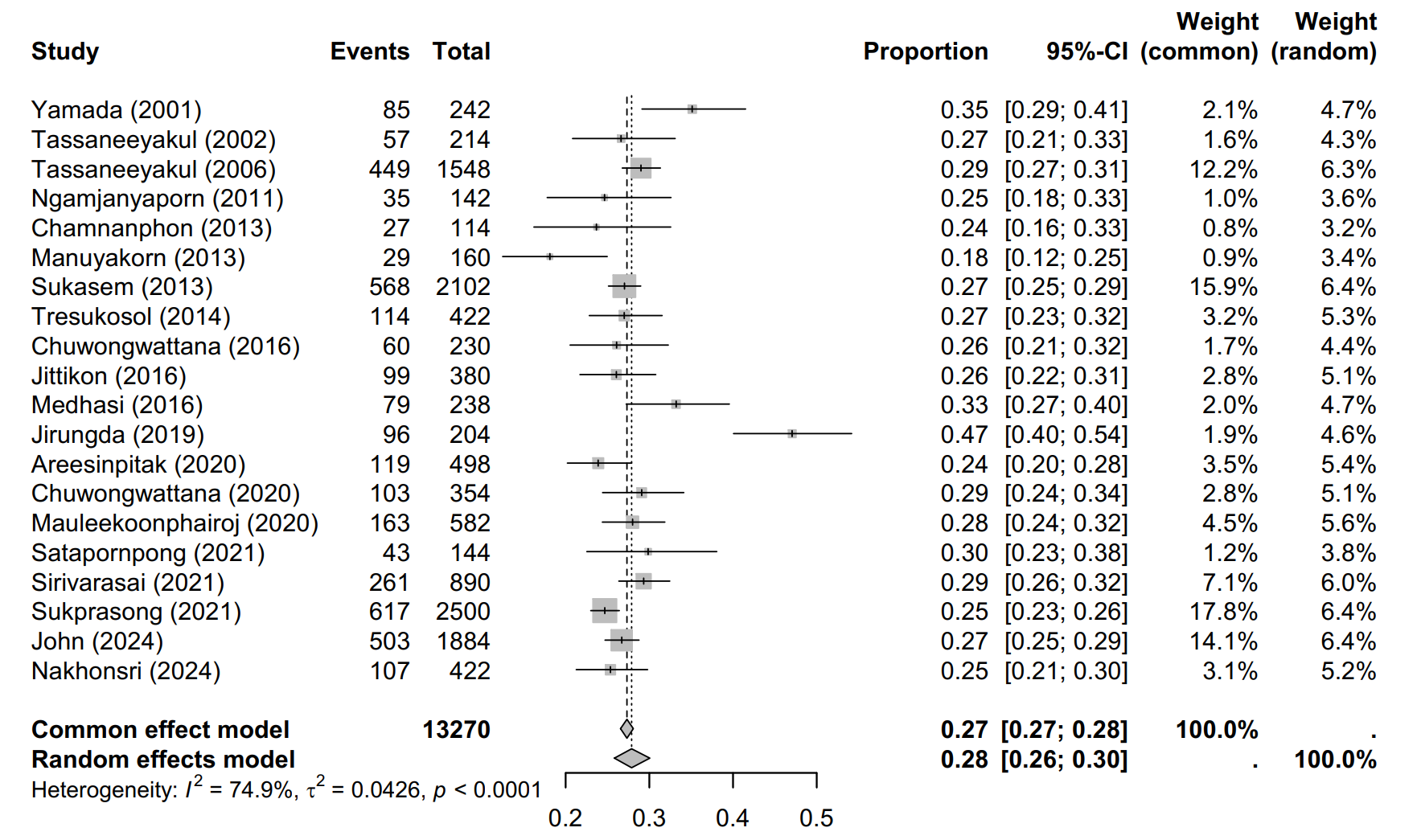


b.


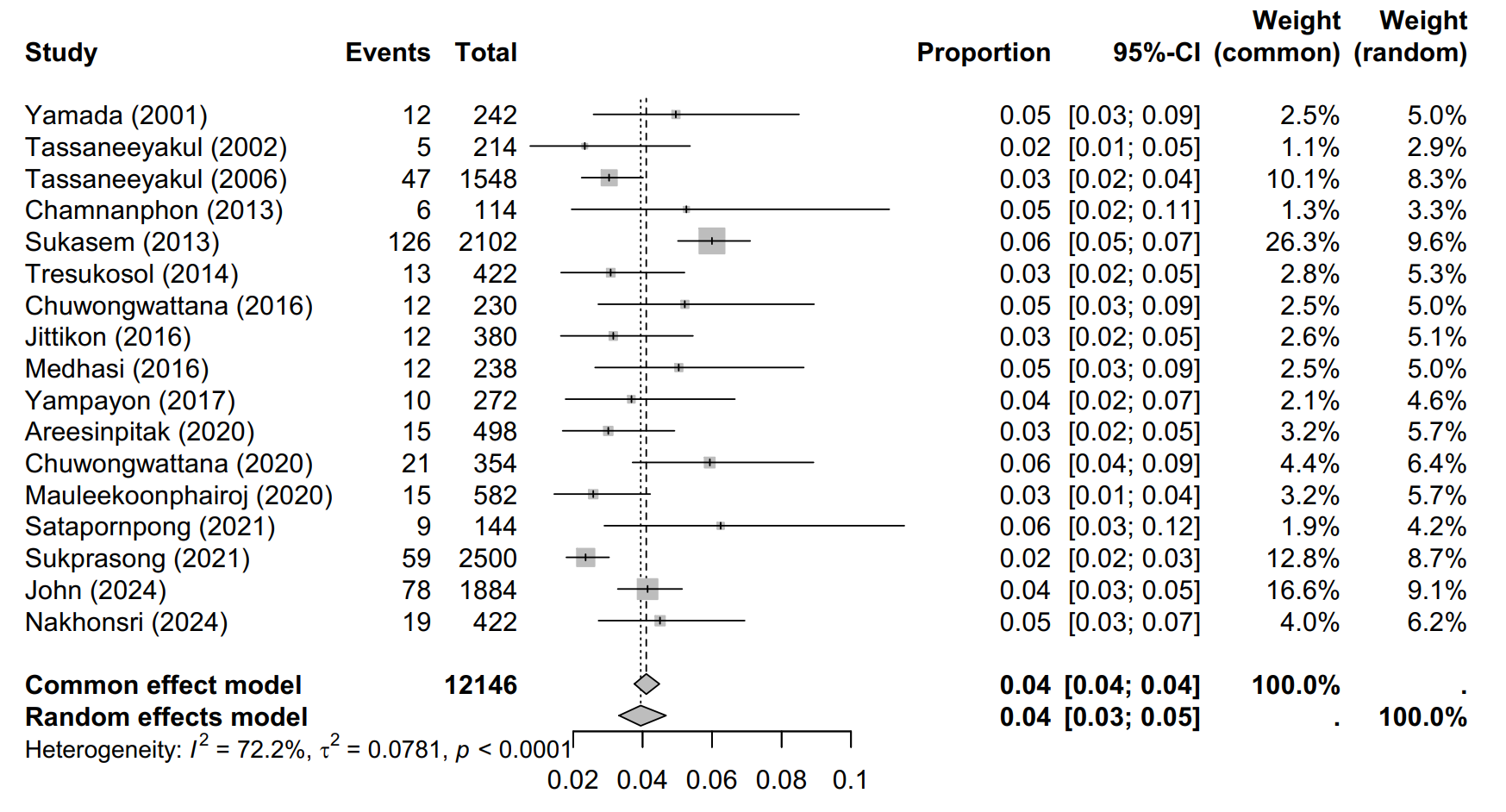


c.


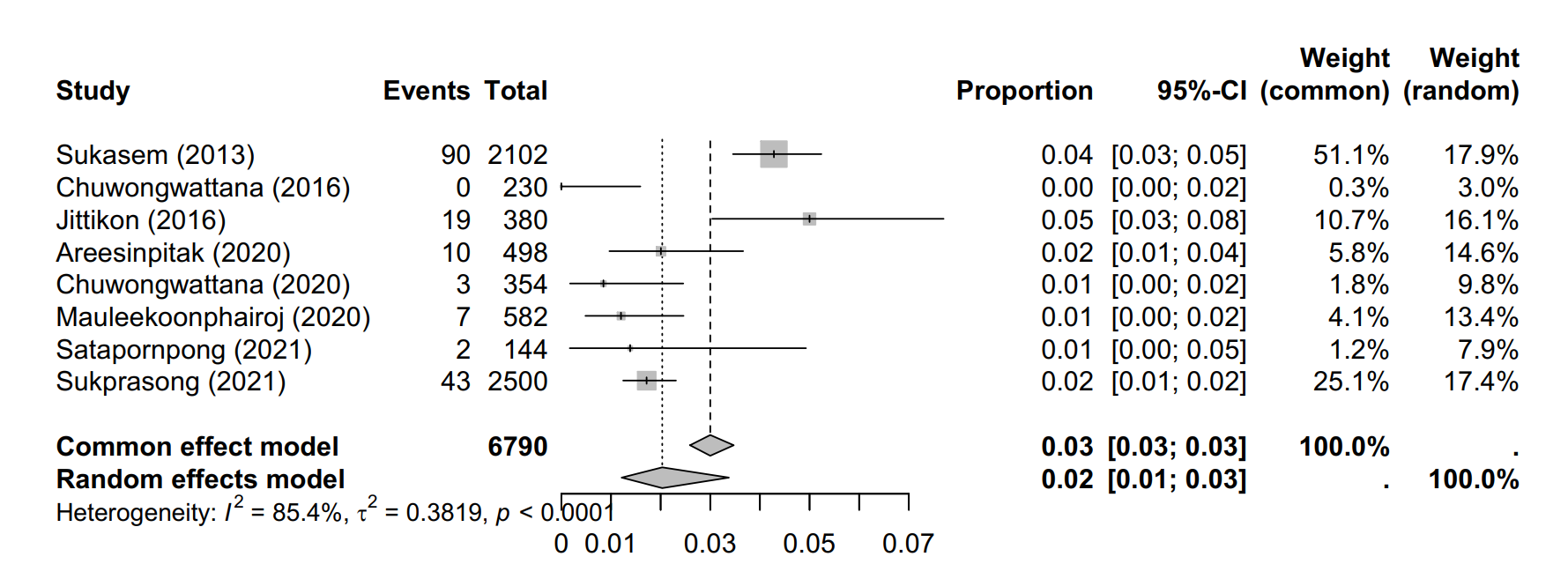


# Supplementary Figure 8. Meta-analysis of allele frequencies in Thailand: (a) for *2 alleles; (b) for *3 alleles; (c) for *17 alleles

a.


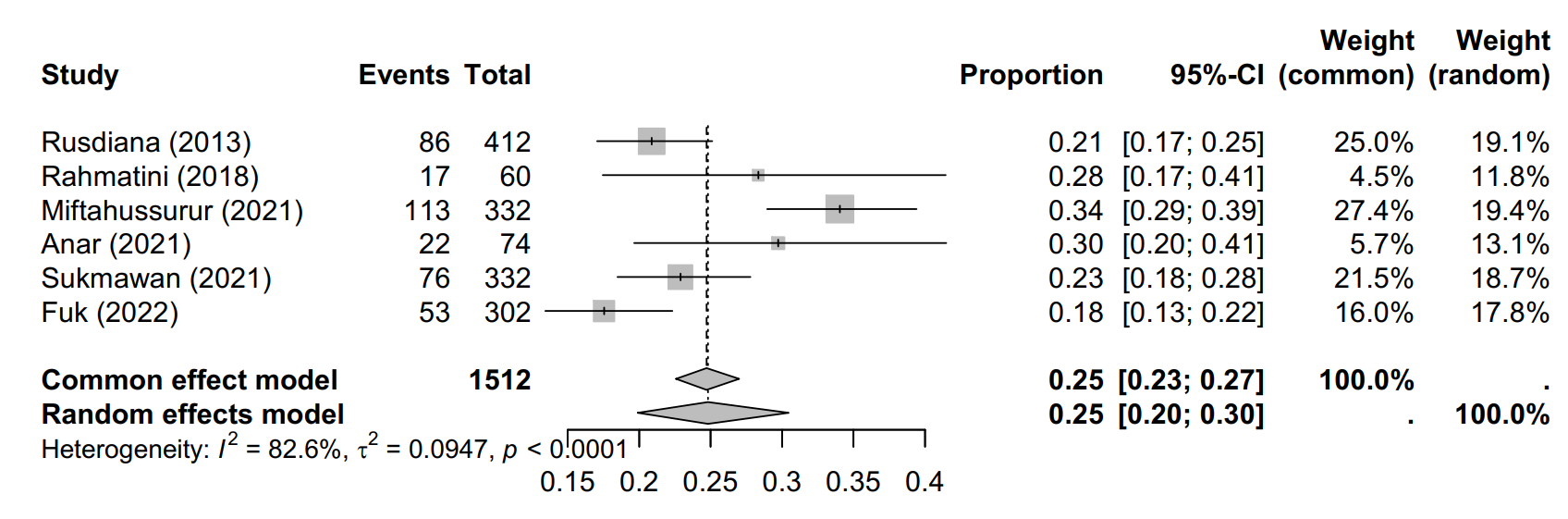


b.


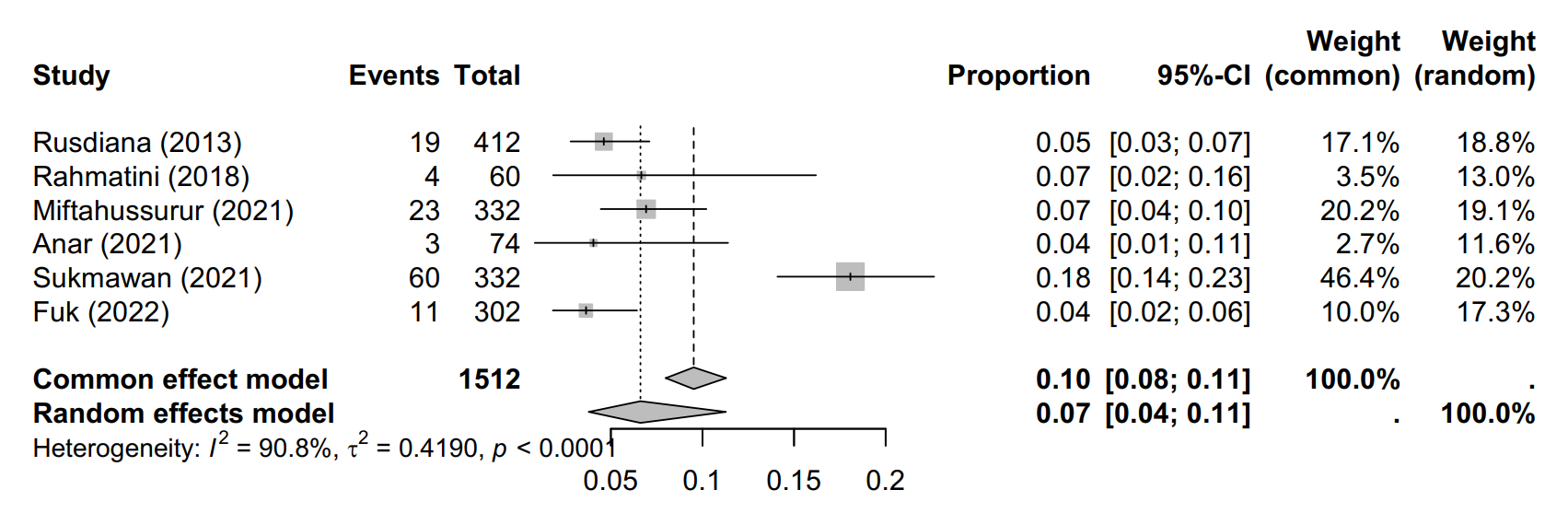


c.


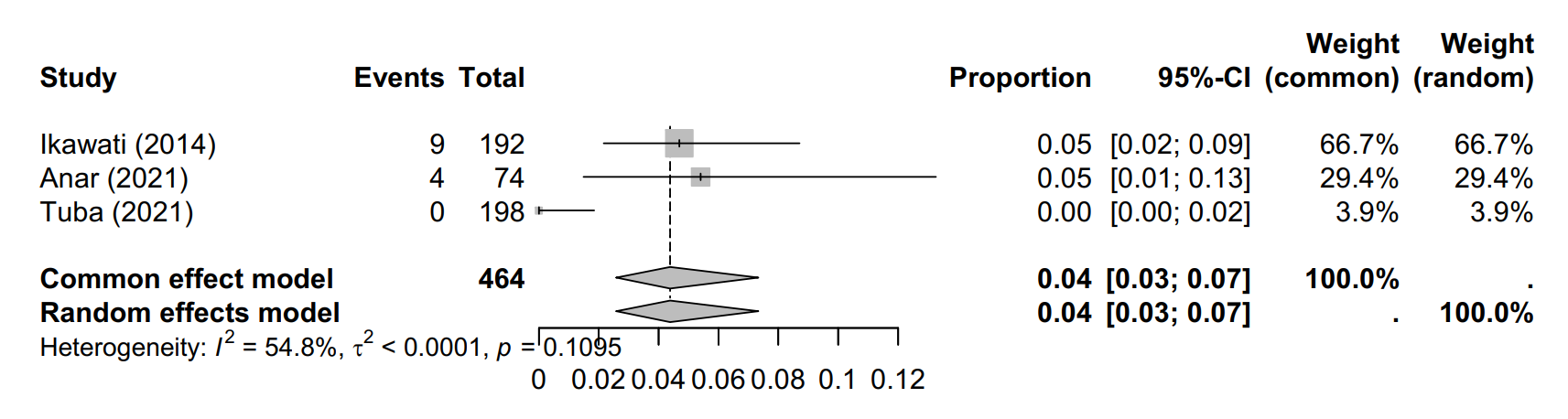


# Supplementary Figure 9. Meta-analysis of allele frequencies in Indonesia: (a) for *2 alleles; (b) for *3 alleles; (c) for *17 alleles

a.


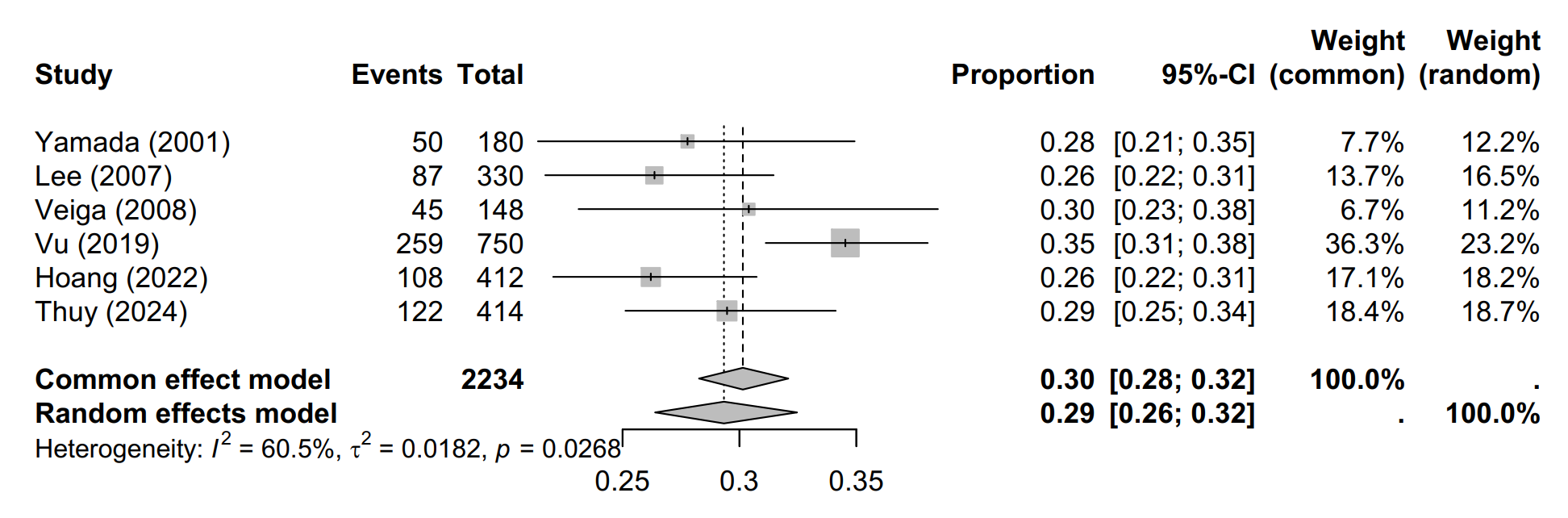


b.


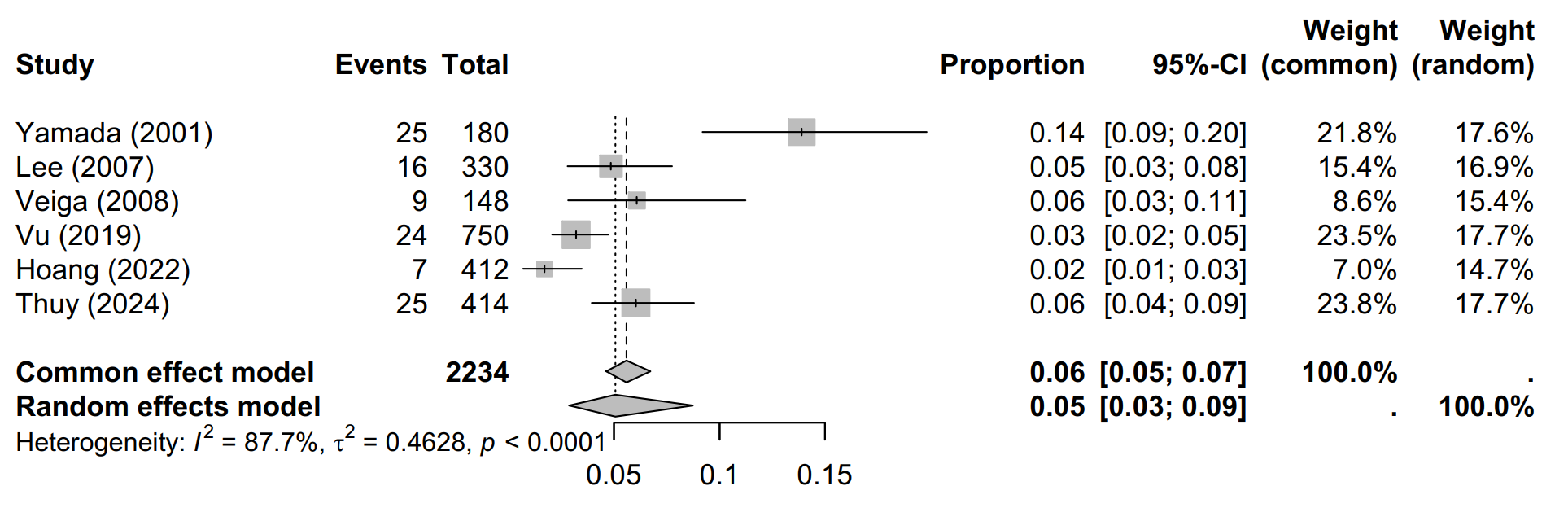


c.


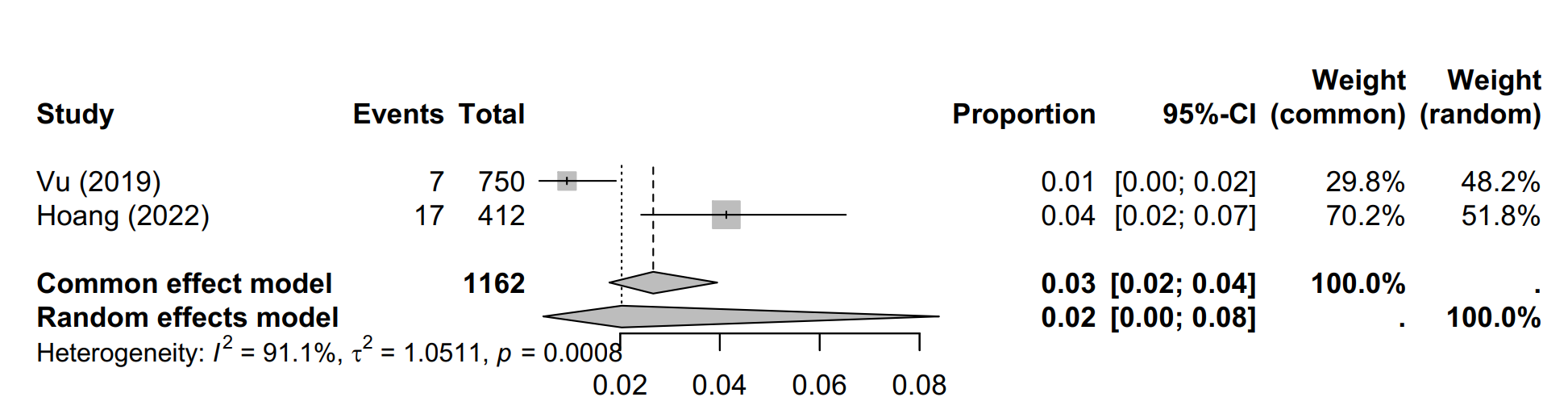


# Supplementary Figure 10. Meta-analysis of allele frequencies in Vietnam: (a) for *2 alleles; (b) for *3 alleles; (c) for *17 alleles

a.


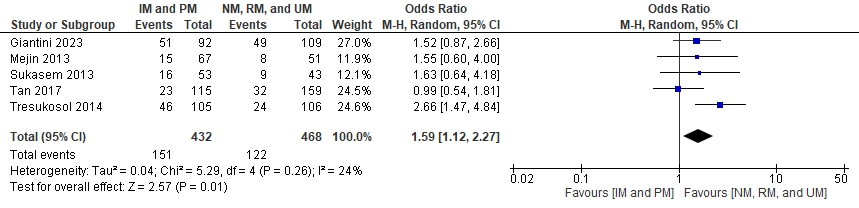


b.


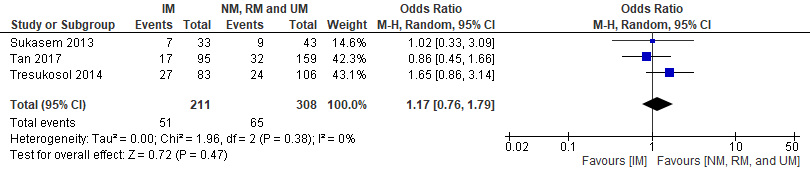


c.


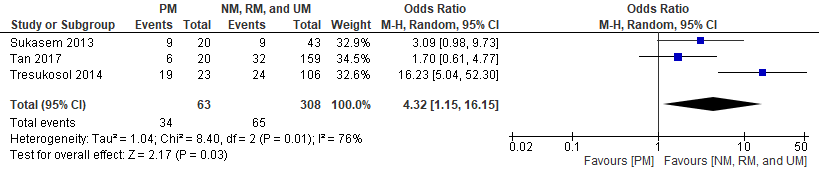


# Supplementary Figure 11. (a) Forest plot comparing the CYP2C19 intermediate metabolizer (IM) and poor metabolizer (PM) phenotypes to the normal metabolizer (NM), rapid metabolizer (RM), and ultra-rapid metabolizer (UM) phenotypes regarding platelet aggregation. (b) Subgroup analysis of IM. (c) Subgroup analysis of PM.

a.


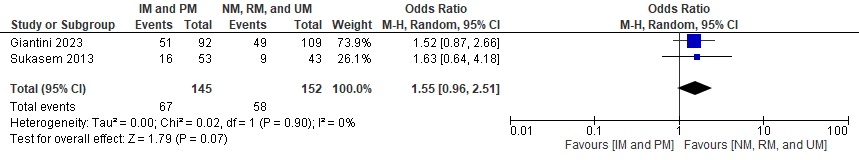


b.


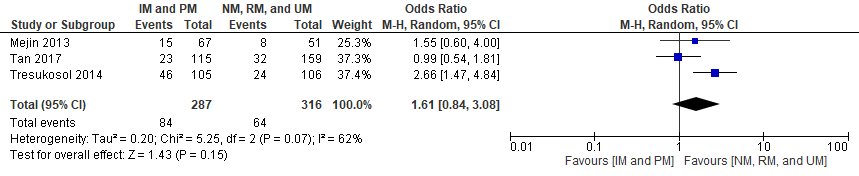


# Supplementary Figure 12. Subgroup analysis of platelet aggregation analysis according to its methodologies. (a) Subgroup analysis of light transmittion aggregation (LTA) method. (b) Subgroup analysis of multiple electrode platelet aggregometry (MEA) method.

a.


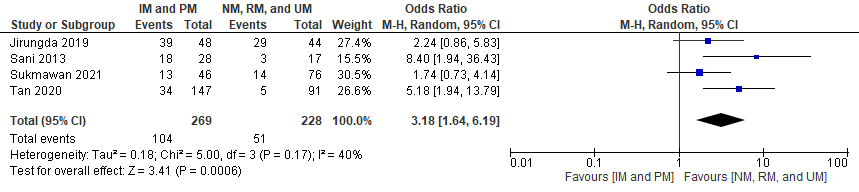


b.


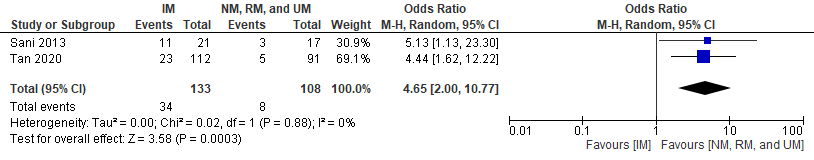


c.


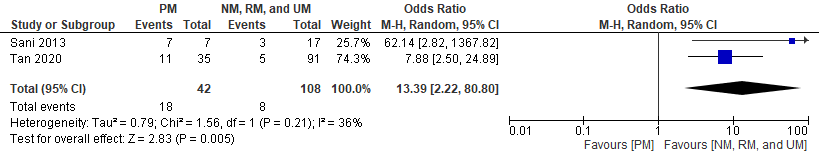


# Supplementary Figure 13. (a) Forest plot comparing the CYP2C19 intermediate metabolizer (IM) and poor metabolizer (PM) phenotypes to the normal metabolizer (NM), rapid metabolizer (RM), and ultra-rapid metabolizer (UM) phenotypes regarding clopidogrel resistance. (b) Subgroup analysis of IM. (c) Subgroup analysis of PM.


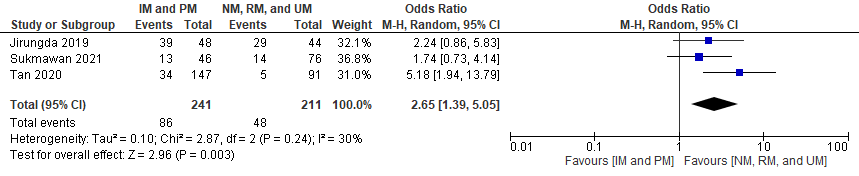


# Supplementary Figure 14. Subgroup analysis of clopidogrel resistance based on VerifyNow^TM^ method


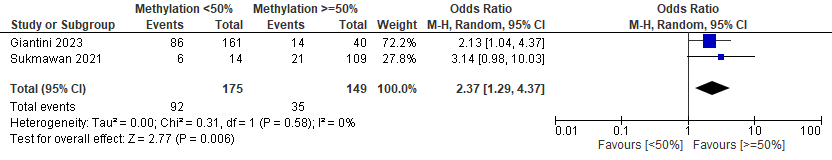


# Supplementary Figure 15. Forest plot comparing the CYP2C19 DNA methylation <50% to >=50% regarding clopidogrel resistance.

a.


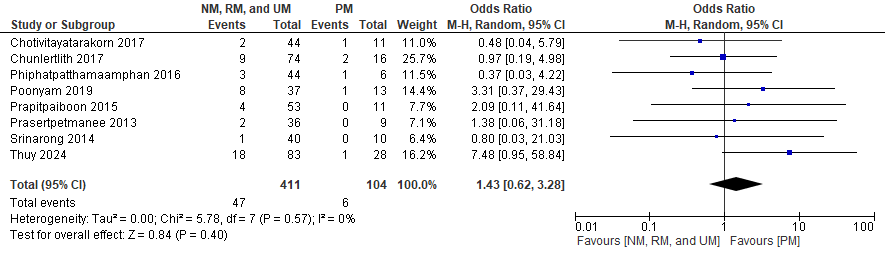


b.


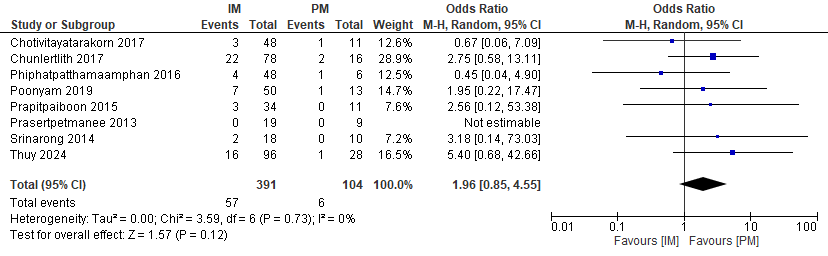


# Supplementary Figure 16. Forest plot comparing CYP2C19 phenotypes of (a) normal metabolizer (NM), rapid metabolizer (RM), and ultrarapid metabolizer (UM) versus poor metabolizer (PM) phenotype, and (b) intermediate metabolizer (IM) versus PM phenotype in relation to treatment failure in *Helicobacter pylori* infection on proton pump inhibitor treatment.

a.


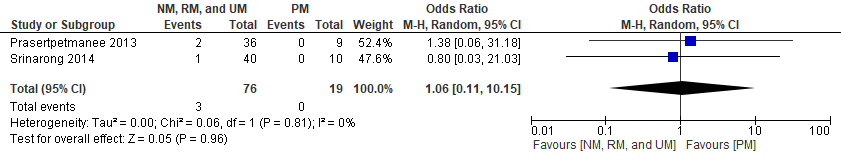


b.


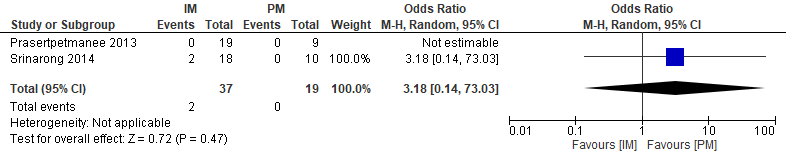


c.


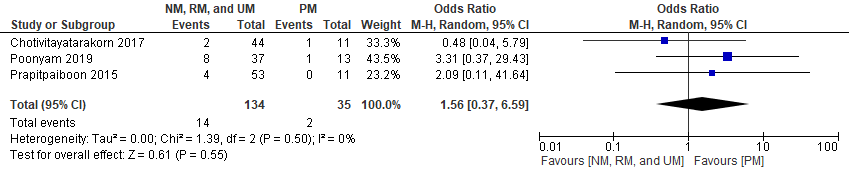


d.


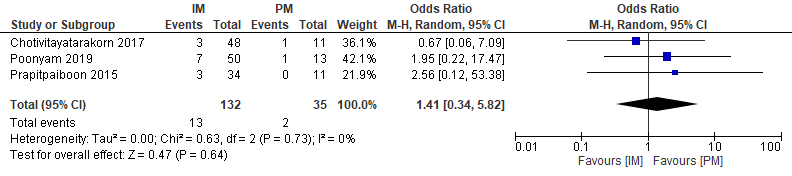


# Supplementary Figure 17. Forest plot comparing CYP2C19 phenotypes of (a) normal metabolizer (NM), rapid metabolizer (RM), and ultrarapid metabolizer (UM) versus poor metabolizer (PM) phenotype, and (b) intermediate metabolizer (IM) versus PM phenotype in relation to treatment failure in *Helicobacter pylori* infection on lansoprazole treatment. (c) NM, RM, UM versus PM phenotype, (d) IM versus PM phenotype on dexlansoprazole treatment
